# Chicken embryonic stem cells and primordial germ cells display different heterochromatic histone marks than their mammalian counterparts

**DOI:** 10.1186/s13072-016-0056-6

**Published:** 2016-02-10

**Authors:** Clémence Kress, Guillaume Montillet, Christian Jean, Aurélie Fuet, Bertrand Pain

**Affiliations:** Inserm, U1208, INRA, USC1361, Stem Cell and Brain Research Institute, 18 avenue du Doyen Lépine, 69500 Bron, France; Université de Lyon, Université Lyon 1, Lyon, France

**Keywords:** Chicken blastocyst, Chicken embryonic stem cells, Chicken primordial germ cells, H3K27me3, DNA methylation, Epigenetic reprogramming

## Abstract

**Background:**

Chromatin epigenetics participate in control of gene expression during metazoan development. DNA methylation and post-translational modifications (PTMs) of histones have been extensively characterised in cell types present in, or derived from, mouse embryos. In embryonic stem cells (ESCs) derived from blastocysts, factors involved in deposition of epigenetic marks regulate properties related to self-renewal and pluripotency. In the germ lineage, changes in histone PTMs and DNA demethylation occur during formation of the primordial germ cells (PGCs) to reset the epigenome of the future gametes. Trimethylation of histone H3 on lysine 27 (H3K27me3) by Polycomb group proteins is involved in several epigenome-remodelling steps, but it remains unclear whether these epigenetic features are conserved in non-mammalian vertebrates. To investigate this question, we compared the abundance and nuclear distribution of the main histone PTMs, 5-methylcytosine (5mC) and 5-hydroxymethylcytosine (5hmC) in chicken ESCs, PGCs and blastodermal cells (BCs) with differentiated chicken ESCs and embryonic fibroblasts. In addition, we analysed the expression of chromatin modifier genes to better understand the establishment and dynamics of chromatin epigenetic profiles.

**Results:**

The nuclear distributions of most PTMs and 5hmC in chicken stem cells were similar to what has been described for mammalian cells. However, unlike mouse pericentric heterochromatin (PCH), chicken ESC PCH contained high levels of trimethylated histone H3 on lysine 27 (H3K27me3). In differentiated chicken cells, PCH was less enriched in H3K27me3 relative to chromatin overall. In PGCs, the H3K27me3 global level was greatly reduced, whereas the H3K9me3 level was elevated. Most chromatin modifier genes known in mammals were expressed in chicken ESCs, PGCs and BCs. Genes presumably involved in de novo DNA methylation were very highly expressed. *DNMT3B* and *HELLS/SMARCA6* were highly expressed in chicken ESCs, PGCs and BCs compared to differentiated chicken ESCs and embryonic fibroblasts, and *DNMT3A* was strongly expressed in ESCs, differentiated ESCs and BCs.

**Conclusions:**

Chicken ESCs and PGCs differ from their mammalian counterparts with respect to H3K27 methylation. High enrichment of H3K27me3 at PCH is specific to pluripotent cells in chicken. Our results demonstrate that the dynamics in chromatin constitution described during mouse development is not universal to all vertebrate species.

**Electronic supplementary material:**

The online version of this article (doi:10.1186/s13072-016-0056-6) contains supplementary material, which is available to authorized users.

## Background

Chromatin epigenetic modifications are important for metazoan development. An extensive body of prior work has thoroughly described two such modifications, DNA methylation and histone post-translational modifications (PTMs), during early mouse development, when they undergo particularly striking changes in abundance and distribution. The demethylation and remethylation of the embryonic genome and the associated chromatin remodelling after fertilisation are the key steps of epigenetic reprogramming during development [1–3]. DNA methylation patterns and histone marks are globally remodelled in primordial germ cells (PGCs) when they migrate to the genital ridges [4–6].

Many aspects of the molecular mechanisms responsible for establishment and removal of the epigenetic marks have been dissected in mouse embryonic stem cells (ESCs) [7]. Because these cells retain their developmental properties after being isolated and established in vitro from the inner cell mass of the blastocyst, they are the archetypal pluripotent cells. ESCs can self-renew in vitro and give rise to all lineages of the developing embryo, including the germ lineage, when injected back into a recipient embryo (for a review, see [8]). PGCs are closely related to ESCs [9]. In mouse, they originate from a few pluripotent proximal epiblast cells, express pluripotency-associated genes and can be used to derive pluripotent embryonic germ (EG) cells [10]. Mouse ESCs exhibit specific chromatin features that are hypothesised to contribute to their pluripotency [11]. As in more differentiated cell types, epigenetic modifications such as acetylation of histone tails, notably on histone H3 lysine 9 (H3K9ac), and methylation of histone H3 lysine 4 (H3K4me) and lysine 36 (H3K36) stabilise the expression of active genes, whereas methylation of DNA at the 5th-position of cytosines (5mC) and histone H3 lysine 9 (H3K9me) or 27 (H3K27me) lock inactive genes in a silenced state. Histone acetyltransferases (HATs) and deacetylases (HDACs), histone lysine methyltransferases (KMTs) and demethylases (KDMs), and DNA methyltransferases (DNMTs) catalyse these modifications together with other chromatin modifiers such as nucleosome remodelling factors of the SWI/SNF family [12].

In the nuclei of mouse ESCs, the majority of chromatin is homogenous, decondensed and devoid of the more condensed heterochromatin observed in differentiated cells [13, 14]. Additionally, undifferentiated ESCs exhibit significant fluidity in their lamin scaffolds, as well as hyperdynamic chromatin exchanges of core histones H2B and H3, and linker histone H1 and heterochromatin protein 1 (HP1), in comparison with more differentiated cells [15, 16]. This less constrained chromatin conformation has been proposed to confer a high degree of plasticity in chromatin-related gene regulation mechanisms, enabling rapid integration of developmental cues [15, 16]. Notably, the promoters of developmental regulator genes that are expressed later are often not condensed in fully repressed chromatin, but rather in a bivalent chromatin state with histone PTMs typical of both active and inactive domains, i.e. trimethylation of histone H3 lysine 4 (H3K4me3) and lysine 27 (H3K27me3), respectively [17, 18].

Trimethylation of H3K27 is catalysed by the EZH1/KMT6B and EZH2/KMT6A methyltransferases, associated with the SUZ12 and EED1 proteins to form the core of the Polycomb repressive complex 2 (PRC2), which interacts with partners such as JARID2 and CDYL involved in repressive chromatin. Methylated H3K27 is bound by CBX proteins from the Polycomb repressive complex 1 (PRC1) in which RING1A/RNF1 or RING1B/RNF2 catalyses H2AK119 ubiquitination (H2AK119ub) [19]. A non-canonical form of PRC1 containing RYBP and KDM2B/FBXL10 together with RING1B/RNF2 deposits H2AK119ub independently of PRC2. H2AK119ub can in turn drive PRC2 recruitment [7]. The Polycomb group (PcG) proteins are essential for cell fate transitions and proper development in mammals [20, 21]. The dynamics of the H3K27me3 mark during mouse development suggests that the H3K27/PcG chromatin repression pathway may operate as a transient repression mechanism, termed facultative heterochromatin and distinct from constitutive heterochromatin [22]. Constitutive heterochromatin domains are repressed by the H3K9me/HP1 pathway reinforced by DNA methylation [21]. These domains notably include pericentric heterochromatin (PCH), the condensed chromatin which embeds DNA repeats of centromeres and pericentromeres of multicellular eucaryotes and forms the cytologically distinct chromocentres [23]. H3K27me3 is transiently enriched at PCH of paternal origin in mouse late zygotes, before it becomes distributed in more euchromatic regions of the nucleus in the blastocyst and in differentiated cells, with accumulation on the inactivated X chromosome in female cells [24, 25]. In mouse PGCs, a global gain in H3K27me3, concomitant with a loss of H3K9me, occurs after specification and before the entry of these migratory cells into the gonads, where epigenetic reprogramming takes place [4, 5]. In mouse ESCs, most of H3K27me3 is found across the body of repressed genes and at bivalent gene promoters [26, 27]. However, the enrichment at developmental gene promoters is greatly reduced when the cells are shifted to the naive state by culture in 2i medium instead of serum [28]. When H3K27 trimethylation is abolished by inactivation of the *EZH1* and *EZH2* genes, ESCs self-renew but exhibit some differentiation defects, likely due to upregulation of PcG targets and failure to extinguish expression of the pluripotency genes *NANOG* and *NR0B1* [29]. Invalidation of other PcG genes also impairs ESC pluripotency by inducing misregulation of lineage-specific genes [21].

The modes of H3K27me/PcG chromatin assembly on target genes are not yet fully understood. One possible targeting mechanism is default assembly, which would be antagonised by counteracting histone modifications or DNA methylation [30–33]. Indeed, in mouse ESCs, the genome methylation level also varies with the level of pluripotency. Maintenance of hypomethylation on the promoters of developmental and housekeeping genes is essential for ESC pluripotency [34, 35]. The action of DNMTs is counterbalanced by the conversion of 5mC to 5-hydroxymethylcytosine (5hmC) by the ten–eleven translocation (TET) enzymes, under the control of the pluripotency factors NANOG and OCT4, and by the presence of PcG proteins [36, 37]. When mouse ESCs are grown in 2i conditions instead of serum-containing medium, their genome contains less 5mC and 5hmC, suggesting that DNA methylation dynamics in cultured ESCs recapitulates early developmental processes [38–40]. The interplay between H3K27me/PcG and DNA methylation may also be at work during PGC expansion and migration. Indeed, PGCs undergo genome demethylation via the 5hmC intermediate before an increase in the level of H3K27me3; these two events may be causally related [4, 5, 41–44].

The characteristics and dynamics of the epigenome during development are evolutionarily conserved between mammalian species, although significant differences are observed among species, notably in regard to DNA methylation patterns and regulatory networks in preimplantation embryos and PGCs [45–47]. In non-mammalian vertebrates such as zebrafish and *Xenopus*, the limited available data indicate that global demethylation of the genome does not occur after fertilisation (reviewed in [48]). Given the heterogeneity of epigenetic regulation between vertebrate species, in order to understand the link between chromatin modifications and pluripotency, it is essential to study additional model organisms with different developmental characteristics. In avian species, the few available data on the epigenome are focused on DNA methylation in chicken germ line [49–51]. The nuclear distributions of several histone PTMs have been investigated in a lymphoblastoid cell line and in somatic cells [52, 53]. As in mammalian cells, these distributions are related to the nuclear positioning of the different chromosomal elements in interphase nuclei. The chicken karyotype comprises ten pairs of macrochromosomes, 28 pairs of microchromosomes and a pair of sex chromosomes (a W and a Z chromosome in the heterogametic females, and two Z chromosomes in males). Constitutive heterochromatin properties are poorly documented for chicken cells. Chicken genome contains about 10–15 % repetitive DNA comprising several families of elements [54, 55]. The most unevenly distributed are the CNM (chicken nuclear membrane) and PO41 (Pattern of 41) tandem repeats, which are found in large arrays of dozens of units covering several kilobases, associated with subtelomeric and centromeric regions  [56]. Centromere DNA sequences from the chicken macrochromosomes, with the exception of chromosome 5, contain chromosome-specific homogeneous tandem repetitive arrays comprising mainly LINE type CR1 (long interspersed elements; chicken repeat 1) transposons. These arrays can span several hundred kilobases, but the CENP-A-associated region spans only about 30 kb, suggesting that these repeats may be the base for PCH assembly adjacent to the kinetochore domain [57]. Centromeres and pericentromeres of minichromosomes contain the majority of CNM repeats, which are also present in the centromere regions of macrochromosomes 6 and 9, and in some non-centromeric clusters of macrochromosomes 3, 6 and 9 [58, 59]. In the already studied chicken cells, (peri) centromere tandem repeats of microchromosomes occupy the DAPI-positive core region of the majority of chromocentres, while kinetochores arrange at chromocentres’ periphery [53], as in mouse cells [23]. The clustering of microchromosomal centromeres establishes common chromocentres located at the peripheral heterochromatin boundary, perinucleolar area, and in the nuclear interior. In contrast, chromosome-specific centromeric tandem repeats and unique sequences in centromere regions of macrochromosomes do not form clusters, are rarely associated with chromocentres and occupy the nuclear periphery [53]. A radial nuclear organisation can also be observed for histone PTMs [52, 53]. H3K9me3 is enriched in the peripheral layer of heterochromatin and in more internally positioned chromocentres. H3K27me3 and H3K9me2 are restricted to the peripheral zone of the nucleus. Whether H3K27me3 distribution varies during chicken early embryonic development as in mammals is not known.

In chicken, ESCs were derived from in vitro cultures of chicken blastodermal cells (BCs) taken from stage X–XII embryos [60], and transcriptome analysis revealed that these long-term cultured cells resemble mouse ESCs in terms of gene expression [61, 62]. In avian species, PGCs that migrate to the genital ridges after being specified early in the epiblast can be isolated from embryonic blood and cultured over the long term without losing their properties [63, 64]. In this study, we compared the major histone PTMs and DNA methylation, as well as the expression of genes encoding chromatin modifiers, between pluripotent stem cells and differentiated cells of chicken. We identified specific chromatin signatures of chicken pluripotent stem cells and showed that several chromatin modifications are similar between chicken ESCs and PGCs and mammalian cells, whereas others differ, notably the distribution of H3K27me3.

## Results

**Fig. 1:**
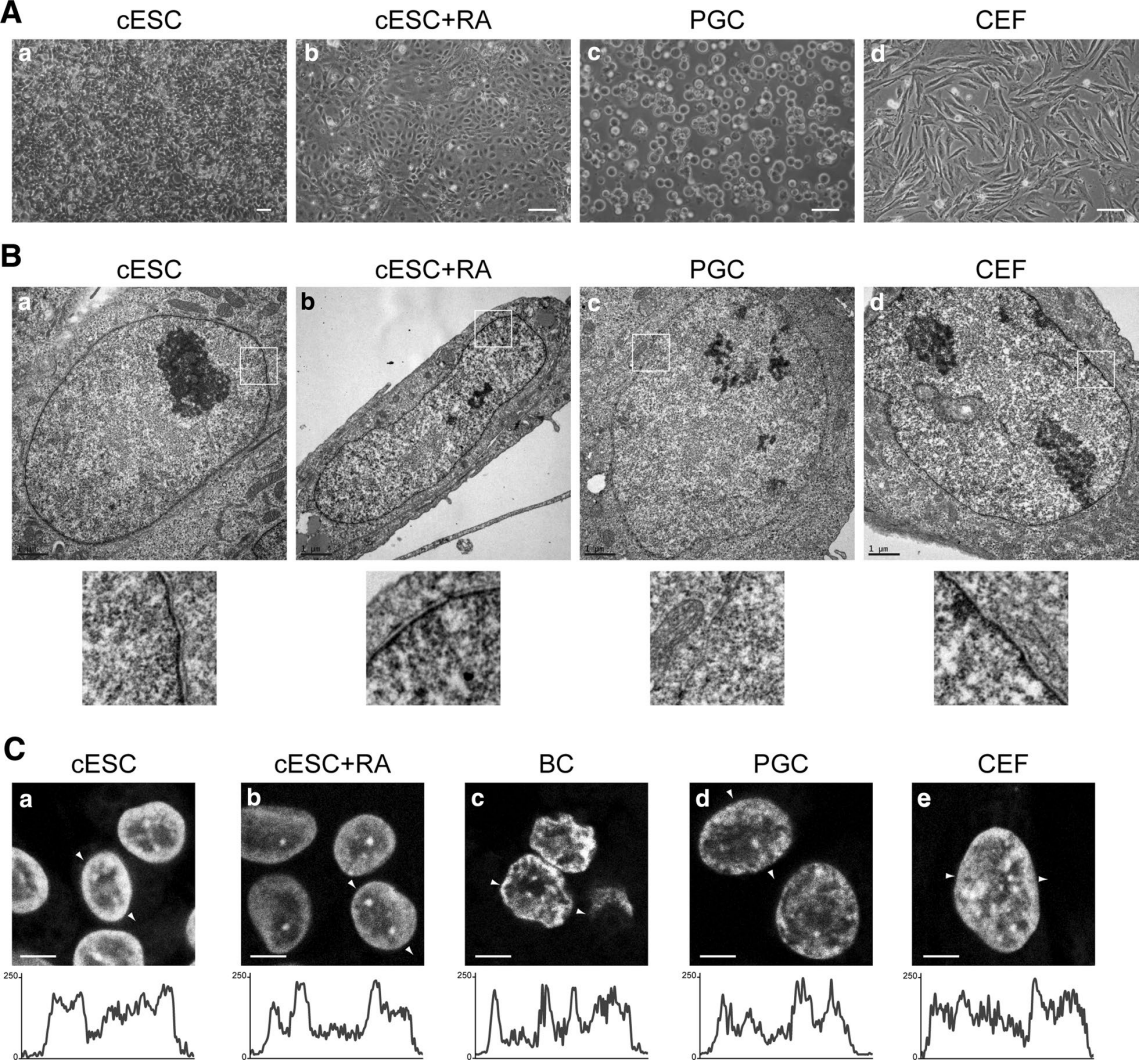
Nuclear morphology of chicken ESCs, RA-differentiated ESCs, BCs, PGCs and CEFs. **A** Light micrographs of cell cultures. Pluripotent chicken embryonic stem cells (cESCs) were grown on STO feeders (**a**) or induced to differentiate (**b**) by retinoic acid (RA) treatment (cESC + RA). Chicken primordial germ cells (PGCs) were derived from embryonic blood and subjected to long-term culture (**c**). Primary chicken embryonic fibroblasts (CEFs) were prepared from 11- to 12-day-old embryos (**d**). *Scale bar* 50 μm. **B** Transmission electron micrographs of nuclei. Zoomed regions (*white boxes*) of the nuclear envelope and associated chromatin are shown below. *Scale bar* 1 μm. **C** DNA staining with TO-PRO-3. Cells were cultured as described in (**A**); blastodermal cells (BCs) were observed in tissue sections from stage X–XII embryos. Single confocal images of representative nuclei are shown. *White arrows* indicate linescan and direction of intensity plots shown below. *Scale bar* 5 μm

To determine whether pluripotency in chicken stem cells is associated with a specific epigenetic state, we analysed different cell types side by side. Chicken ESCs from independent isolates were either maintained under proliferative conditions (Fig. 1Aa) or induced to differentiate by removal of cytokines and growth factors combined with a retinoic acid (RA) treatment for 5 days. Loss of expression of pluripotency-associated genes and induction of differentiation markers [65, 66], as well as drastic proliferation and morphological changes, were observed under these culture conditions (Fig. 1Ab). Chicken ESCs were compared to BCs directly observed in stage X–XII embryo sections, and to PGCs established as long-term cultures (Fig. 1Ac). Finally, chicken embryonic fibroblasts (CEFs) from 12-day-old chicken embryos were cultured for a few passages before analysis (Fig. 1Ad).

### Morphology and ultrastructure of nuclei

First, we examined proliferating and RA-differentiated ESCs, PGCs, and CEFs by transmission electron microscopy (Fig. 1B). Nucleoli were large and generally located in the centre of nuclei in all cell types, and were more expanded in PGCs, which had the highest nucleocytoplasmic ratio among the cell types analysed. In all cell types, chromatin was homogeneously distributed in the nucleoplasm, without large zones of electron-dense heterochromatin. The nucleoplasm was more uniform in undifferentiated ESCs (Fig. 1Ba), and especially in PGCs (Fig. 1Bc) than in RA-differentiated ESCs (Fig. 1Bb) and CEFs (Fig. 1Bd), in which local aggregates of dense chromatin were larger. A discernible layer of dense chromatin, which could be seen just below the nuclear envelope in all other cell types, was absent in PGCs (magnifications in Fig. 1B).

**Fig. 2:**
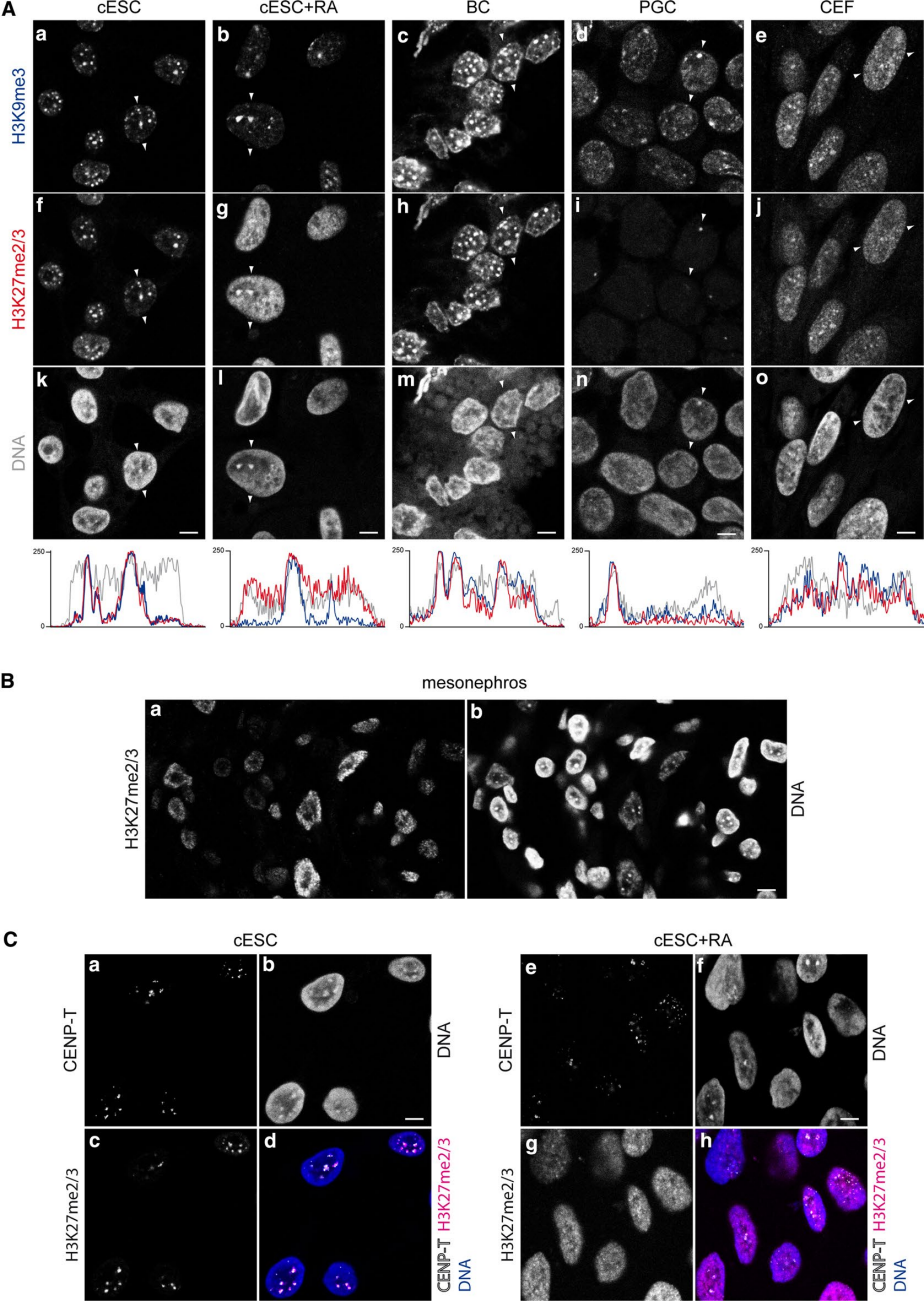
Histone post-translational modifications of pericentric heterochromatin in chicken cells. **A** Co-immunodetection of H3K9me3 (**a**–**e**) and H3K27me2/3 (**f**–**j**), and DNA counterstaining with TO-PRO-3 (**k**–**o**) in nuclei of cESCs, RA-differentiated cESCs, BCs, PGCs and CEFs. *White arrows* indicate linescan and direction of intensity plots below, showing signal for H3K9me3 (*blue*), H3K27me2/3 (*red*) and TO-PRO-3 (*grey*) in the equatorial section of a typical nucleus. **B** Immunodetection of H3K27me2/3 (**a**) and DNA counterstaining by TO-PRO-3 (**b**) in nuclei in tissue sections from 9 day embryonic mesonephros. **C** Co-immunodetection of CENP-T (**a**, **e**) and H3K27me2/3 (**c**, **g**), and DNA counterstaining with TO-PRO-3 (**b**, **f**) in nuclei of cESCs and RA-differentiated cESCs. Overlay of CENP-T (*white*), H3K27me2/3 (*magenta*) and TO-PRO-3 (*blue*) is also shown (**d**, **h**). Single confocal sections of representative nuclei are shown. *Scale bar* 5 μm

We obtained a second overview of chromatin arrangement by staining DNA with the TO-PRO-3 intercalating dye (Fig. 1C). In ESCs, the most intensely stained chromatin regions were arranged in a thick rim at the nuclear periphery, and a few large foci were often observed in the vicinity of the nucleoli or embedded in the peripheral rim (Fig. 1Ca). Thus, large-scale chromatin domains in ESCs form peripheral heterochromatin and chromocentres, as observed in most somatic cell types [53]. The identity of the TO-PRO-3-positive aggregates as chromocentres was confirmed by their proximity to centromeres and their H3K9me3 enrichment (Fig. 2A, C). Upon differentiation with RA, the general profile remained the same, but the chromocentres, notably those located in the nuclear interior, were more intensely stained than the nuclear periphery (Fig. 1Cb). The DNA staining pattern of BCs (from the epiblast) was similar to that of ESCs (Fig. 1Cc). In PGCs, the chromocentres were less easy to identify because the contrast between zones of high- and low-DNA staining was weaker (Fig. 1Cd). The nuclei of CEFs contained numerous small regions of contrasting DNA staining, among which small chromocentres were sometimes discernible (Fig. 1Ce).

### Nuclear distribution of histone PTMs

Next, we investigated whether histone modifications typical of the different chromatin types in mammals were conserved in chicken. The various types of heterochromatin were probed by immunodetection of H3K9me3, H3K9me2 and H3K27me2/3. In ESCs, H3K9me3 was concentrated in large, clearly delimited regions distributed throughout the nucleus (Fig. 2Aa). These regions overlapped with the domains detected using CREST antisera which recognise mammalian centromeric proteins and chicken chromocentres [52], and were in close proximity with kinetochores (see Additional file 1 and below). They were thus identified as chromocentres consisting of PCH enriched in H3K9me3, as previously described in somatic chicken cells. In RA-differentiated ESCs, the H3K9me3 domains were still visible in most nuclei (Fig. 2Ab). They were less numerous and often bigger than in undifferentiated ESC nuclei, probably because of a more frequent clustering of centromeres (Fig. 2C). In BCs, the heterochromatin domains were distributed in large regions similar to those of ESCs, but with the additional presence of perinuclear domains (Fig. 2Ac). H3K9me3-containing domains were also present in PGC nuclei, where they were less clearly delimited than in ESCs (Fig. 2Ad). In CEFs, these domains mostly consisted of small foci; nuclei containing large, easily identifiable chromocentres with high enrichment of H3K9me3 content over the nucleoplasm background were rare (Fig. 2Ae).

Surprisingly, H3K27me2/3 was extensively colocalised with H3K9me3 in ESCs (Fig. 2Af). The colocalisation with H3K9me3 was lost upon RA-induced differentiation (Fig. 2Ag). Indeed, although domains enriched in both H3K9me3 and H3K27me2/3 were still present, the differentiated cell nuclei also contained domains enriched in only one of these histone marks. The global H3K27me2/3 level appeared higher after differentiation, due to the increase in enrichment in the nucleoplasm relative to the chromocentres. This unusual H3K27me2/3 pattern and its dynamics upon RA induction were observed in the five studied ESC isolates. To confirm that H3K27me2/3 was indeed enriched at PCH in chicken ESCs, co-detection was performed using an antibody directed against chicken CENP-T, a component of the inner kinetochore  [57]. This enabled a more precise localisation of the centromere than with the CREST antibody, which recognises foci of CENP-A, B or C in mammalian cells but appears to recognise larger pericentric chromatin domains in chicken cells (Additional file 1). Examination of nuclei in 3D showed that the H3K27me2/3-enriched domains of ESCs were all adjacent to one or several CENP-T spots (Fig. 2C) and thus demonstrated that these H3K27me2/3-and K9me3-rich chromocentres are clusters of PCH. In BCs, H3K27me2/3 was extensively colocalised with H3K9me3 (Fig. 2Ah); in comparison with ESCs, the two marks were more abundant in zones not corresponding to chromocentres. In PGCs, H3K27me2/3 could barely be detected by immunofluorescence, with the exception of one dot in about 50 % of nuclei (Fig. 2Ai). This dot overlapped with a domain strongly enriched in H3K9me3 (Fig. 2Ad) that was brightly counterstained by the DNA dye (Fig. 2An). This large spot may be the chromocentre of W sex chromosome, one of the most massive chromocentres [53]. In CEFs, the H3K27me2/3 distribution pattern was roughly similar to that of H3K9me3, but in a more dispersed fashion when compared with the ESCs and BCs (Fig. 2Aj); in CEFs of higher passage number, the H3K27me2/3 enrichment at PCH tended to be lost (not shown). Additional immunodetection experiments on 9-day-old embryonic tissues, as exemplified here by mesonephros tissue (Fig. 2B), revealed that although the H3K27me2/3 level depended on the cell type, this modification was not enriched at PCH, but was instead dispersed in small foci with a pattern similar to that observed in mouse ESC (see Additional file 2). Immunodetection with an antibody specific to trimethylated H3K27 showed the same distribution patterns than those observed for H3K27me2/3, and competition assays with a methylated peptide confirmed that the observed signal actually indicated the presence of H3K27me3 (see Additional file 3).

**Fig. 3:**
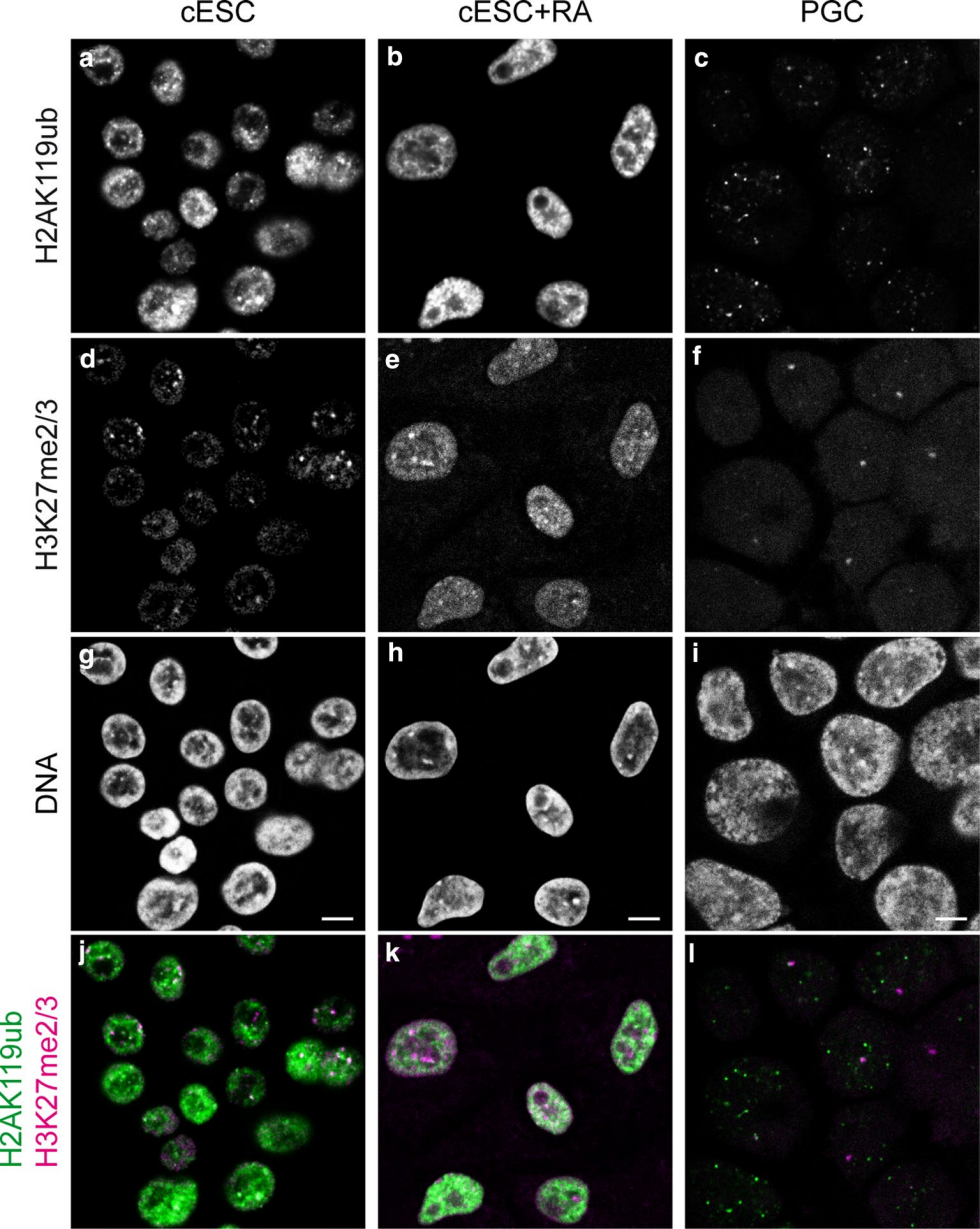
Nuclear distribution of Polycomb group-deposited histone post-translational modifications in chicken ESCs and PGCs. Co-immunodetection of H2K119ub (**a**–**c**) and H3K27me2/3 (**d**–**f**), and DNA counterstaining with TO-PRO-3 (**g**–**i**) in nuclei of cESCs, RA-differentiated cESCs and PGCs. Overlay of H2K119ub (*green*) and H3K27me2/3 (*magenta*) is shown below (**j**–**l**). Single confocal images of representative nuclei are shown. *Scale bar* 5 μm

We examined a second typical mark of Polycomb repression, i.e. H2AK119ub deposited by PRC1. In ESCs, H2AK119ub was mostly present as numerous small foci in nuclear zones that were faintly stained with the DNA dye, i.e. euchromatic regions (Fig. 3a). We also observed a few larger domains, more frequently overlapping with DNA-dense regions and H3K27me2/3 domains (Fig. 3d and j). In RA-differentiated ESCs, H2AK119ub was also present mostly in euchromatic regions, with less highly enriched foci than in ESCs (Fig. 3b). H2AK119ub and H3K27me2/3 did not colocalise; the large, centrally located and H3K27me2/3-enriched chromocentres were often depleted in H2AK119ub (Fig. 3e and k). In PGCs, the nucleoplasm was poor in H2AK119ub, apart from 8–10 foci per cell (Fig. 3c); the large H3K27me2/3 domain present in some nuclei was sometimes adjacent to or overlapping with one of these foci (Fig. 3f and l). Together, the distribution patterns of H2AK119ub in the three cell types suggested that, in general, H3K27me2/3 and H2AK119ub are not enriched in the same chromatin domains, as in mouse ESCs (see Additional file 2).

**Fig. 4:**
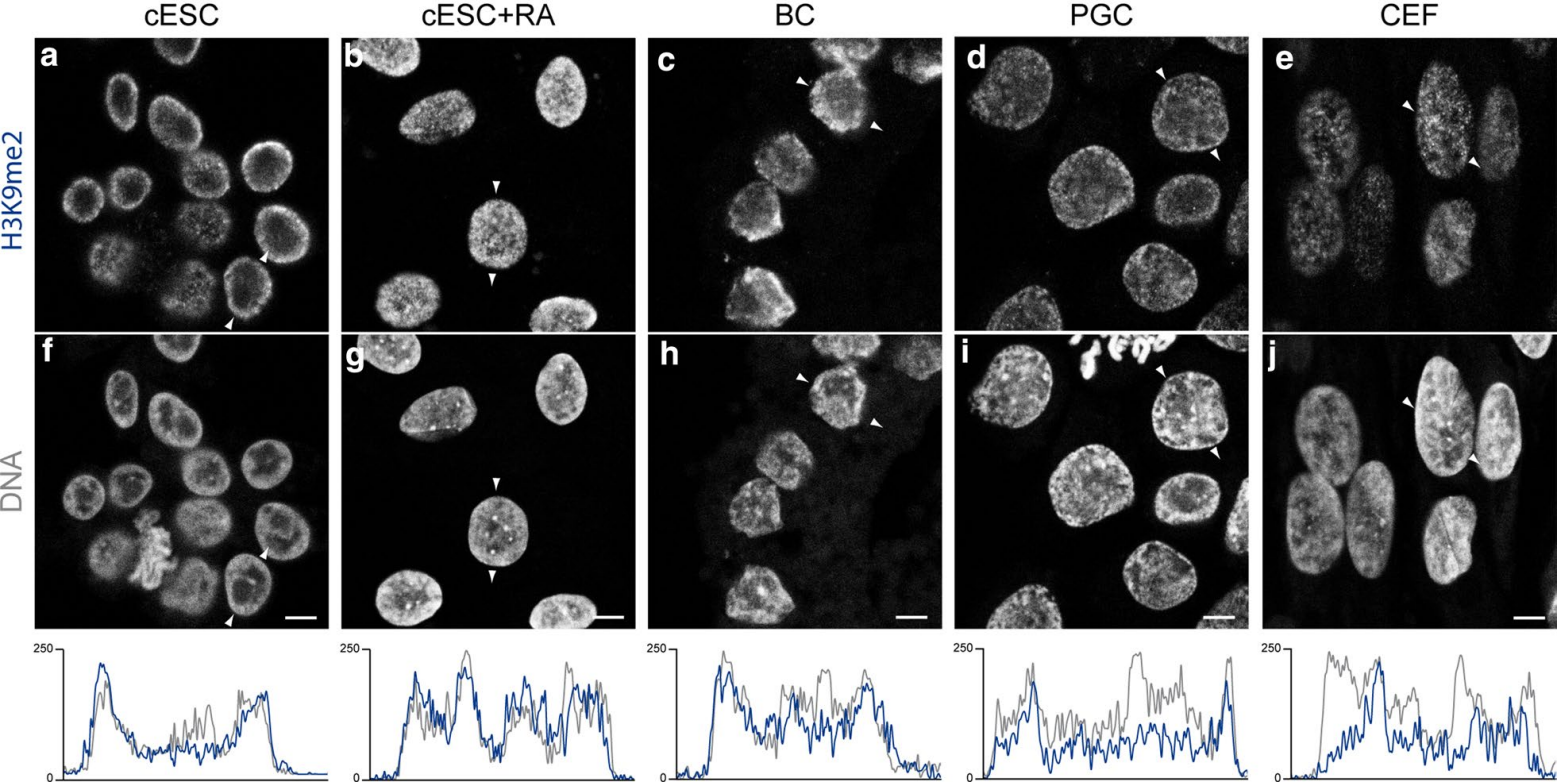
Nuclear distribution of H3K9me2 in chicken ESCs, RA-differentiated ESCs, BCs, PGCs and CEFs. Immunodetection of H3K9me2 (**a**–**e**) and DNA counterstaining with TO-PRO-3 (**f**–**j**) in nuclei of cESCs, RA-differentiated cESCs, BCs, PGCs and CEF. *White arrows* indicate linescan and direction of intensity plots below, showing signal for H3K9me2 (*blue*) and TO-PRO-3 (*grey*) in the equatorial section of a typical nucleus. Single confocal images of representative nuclei are shown. *Scale bar* 5 μm

In all cell types, H3K9me2 was present as numerous tiny foci (Fig. 4). In ESCs (Fig. 4a), BCs (Fig. 4c) and PGCs (Fig. 4d), the foci were mostly in DNA-dense regions at the nuclear periphery but not at chromocentres. In RA-differentiated ESCs (Fig. 4b) and CEFs (Fig. 4e), some of the chromocentres were enriched in H3K9me2. H3K9me2 distribution in chicken cells was thus very similar to what was observed in mouse ESCs (see Additional file 2).

To visualise active chromatin domains, we investigated the distributions of H3K4me3, which is concentrated at active gene regulatory regions, and H3K9ac, a marker of open chromatin. In all cell types analysed, H3K4me3 was distributed in small foci located in nuclear zones that were poorly stained by the DNA dye, i.e. euchromatic zones (see Additional file 4). H3K9ac also formed foci in euchromatic regions, with a less continuous pattern than H3K4me3 (Additional file 4). Thus, chicken ESCs and PGCs are not different from other cell types concerning the location of active chromatin domains in the nucleus.

### Global levels of histone H3 PTMs

**Fig. 5:**
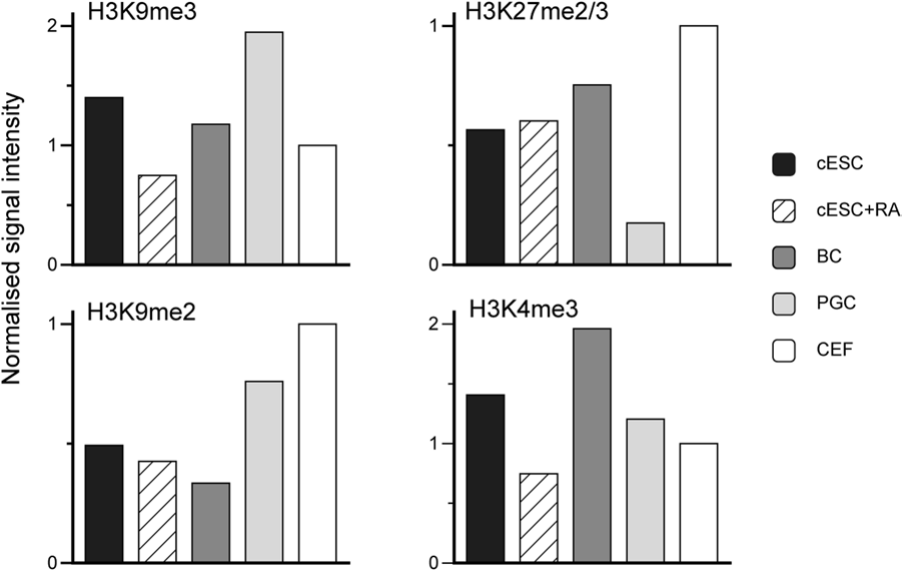
Global histone H3 post-translational modifications levels in chicken ESCs, RA-differentiated ESCs, BCs, PGCs and CEFs. H3K9me3, H3K27me2/3, H3K9me2 and H3K4me3 levels were quantified and normalised to total H3 levels by Western blot analysis of 3 μg of purified histones. Mean values from at least two technical repeats are shown (see “Results” section for details)

Next, we quantified the abundance of histone H3 bearing different PTMs specific for various chromatin types using Western blots. For the H3 modifications that we studied, the levels in the analysed five independent ESC isolates were similar to those observed in BCs (Fig. 5, Additional file 5 and data not shown). H3K9me3 clearly decreased (2–3-fold) when the cells were induced to differentiate by RA, irrespective of the ESC isolate. On the contrary, H3K27me2/3 abundance, high in CEFs, tended to stay similar following RA-induced differentiation. H3K9me2 and H3K4me3 levels in pluripotent and RA-differentiated ESCs were more variable among the different ESC isolates, with a global decrease of H3K9me2 and a more variable trend for H3K4me3 following induction of differentiation. Interestingly, H3K9me3 and H3K9me2 were enriched in PGCs in comparison with ESCs and BCs, whereas H3K27me2/3 level was much lower.

### DNA methylation and hydroxymethylation

**Fig. 6:**
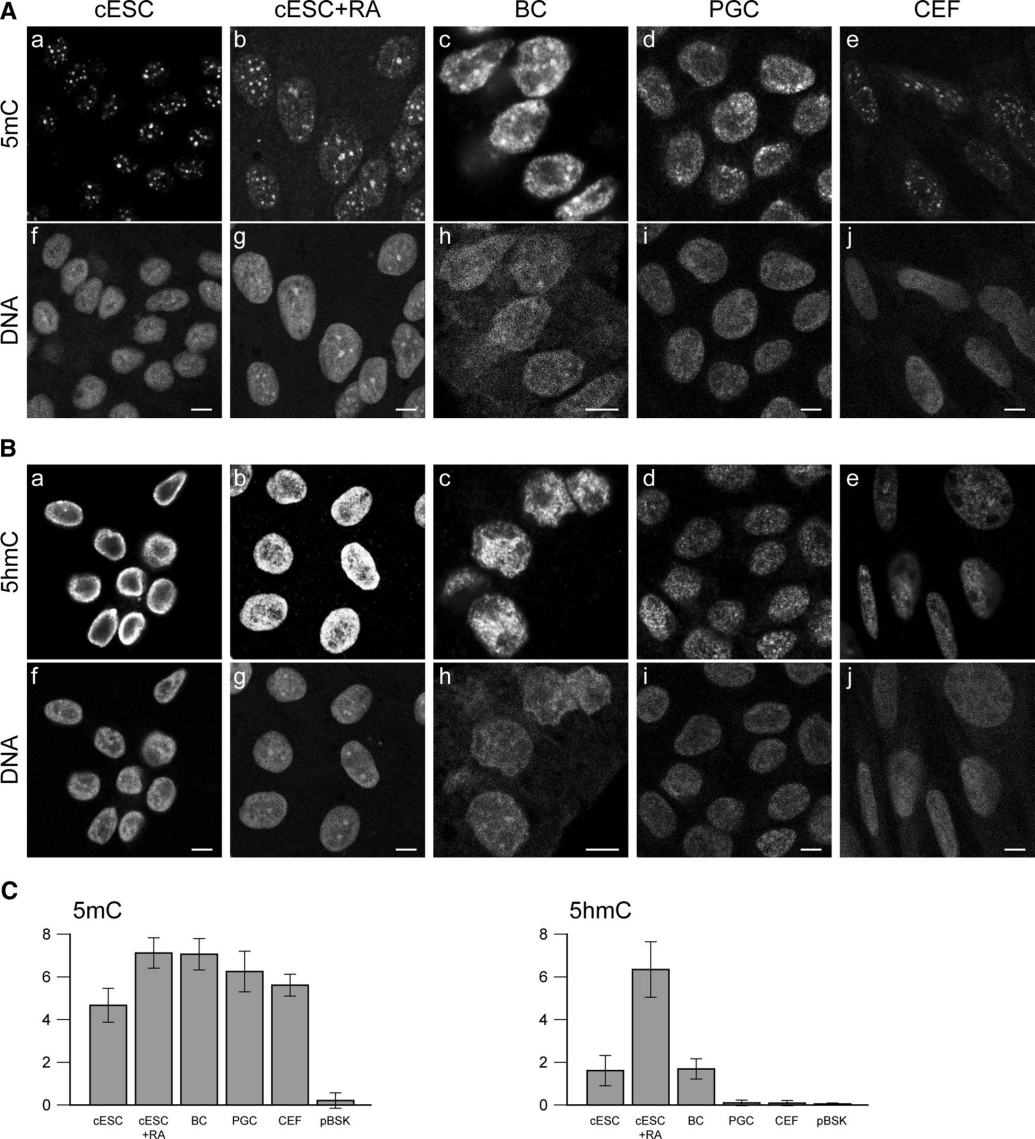
DNA methylation and hydroxymethylation in chicken ESCs, RA-differentiated ESCs, BCs, PGCs and CEFs. **A** Immunodetection of 5-methylcytosine (**a**–**e**) and DNA counterstaining with propidium iodide (**f**–**j**) in nuclei of ESCs, RA-differentiated ESCs, BCs, PGCs and CEFs. **B** Immunodetection of 5-hydroxymethylcytosine (**a**–**e**) and DNA counterstaining with propidium iodide (**f**–**j**) in nuclei of ESCs, RA-differentiated ESCs, BCs, PGCs and CEFs. Single confocal images of representative nuclei are shown. *Scale bar* 5 μm. **C** Global levels of 5-methylcytosine and 5-hydroxymethylcytosine in chicken cells. 5mC and 5hmC levels were quantified by dot blot analysis of 125 ng of denatured genomic DNA from the different cell types; pBSK plasmid was used as a negative control. *Error bars* indicate the standard deviation of the mean signal for three technical replicates of one representative experiment

Immunodetection of 5mC in ESC nuclei showed the presence of foci at chromocentres (Fig. 6Aa), as in mouse ESCs (Additional file 2); these foci were still observed after RA-induced differentiation (Fig. 6Ab). In BCs (Fig. 6Ac) and PGCs (Fig. 6Ad), 5mC domains colocalising with dense chromatin domains could also be seen, but they were more heterogeneous in size and intensity, and less clearly delimited. In CEFs, 5mC formed small foci at chromocentres (Fig. 6Ae). As in mouse ESCs (Additional file 2), the distribution pattern of 5hmC was different: regardless of cell type, 5hmC was not preferentially located in heterochromatic regions. Instead, it formed numerous small foci mostly in euchromatic regions, resulting in an apparently continuous domain in nuclei in which the density was very high, i.e. ESCs (Fig. 6Ba), RA-differentiated ESCs (Fig. 6Bb) and BCs (Fig. 6Bc). In PGCs (Fig. 6Bd) and CEFs (Fig. 6Be), the foci were less dense.

To get a better estimate of the global levels of 5mC and 5hmC in the genome, we quantified the two modifications in genomic DNA prepared from ESCs, RA-differentiated ESCs, BCs, PGCs and CEFs using dot blots (Fig. 6C). Genomic DNA purified from mouse ESCs cultured with 2i or serum, or from mouse EpiSCs was used to validate that the method could detect differences in 5mC and 5hmC contents (Additional file 5). In most isolates among the five studied isolates of chicken ESCs, the level of 5mC was similar to that in the other chicken cell types examined, i.e. RA-differentiated cells, BCs, PGCs and CEFs. In some isolates, we observed a lower level of 5mC in undifferentiated cells (Fig. 6 and data not shown). The 5hmC level was identical in chicken ESCs and BCs. Differentiation by RA resulted in an increase in the 5hmC level in some ESC isolates (Fig. 6 and data not shown). Remarkably, the level of this modification was very low in PGCs and CEFs. Together, these findings indicate that cultured chicken ESCs retain the 5mC and 5hmC levels of BCs.

### Expression of chromatin modifiers

To understand how the histone and DNA marks observed in pluripotent chicken cells are established, we examined the expression of the main chromatin modifiers. Genes encoding chromatin regulators were identified in the chicken genome on the basis of the function of the orthologous genes in the mouse, with special attention to their roles in pluripotency and differentiation. We quantified mRNA levels by RT-qPCR in ESCs, RA-differentiated ESCs, BCs, PGCs and CEFs as a somatic cell type. However, because CEFs proliferate slowly, differences in expression levels between ESCs and CEFs may be linked to proliferation status rather than pluripotency. Consequently, we also analysed monocytic progenitor BM2 cells (data not shown) as proliferating non-pluripotent cells, as done previously for transcriptome analyses [62].

**Fig. 7:**
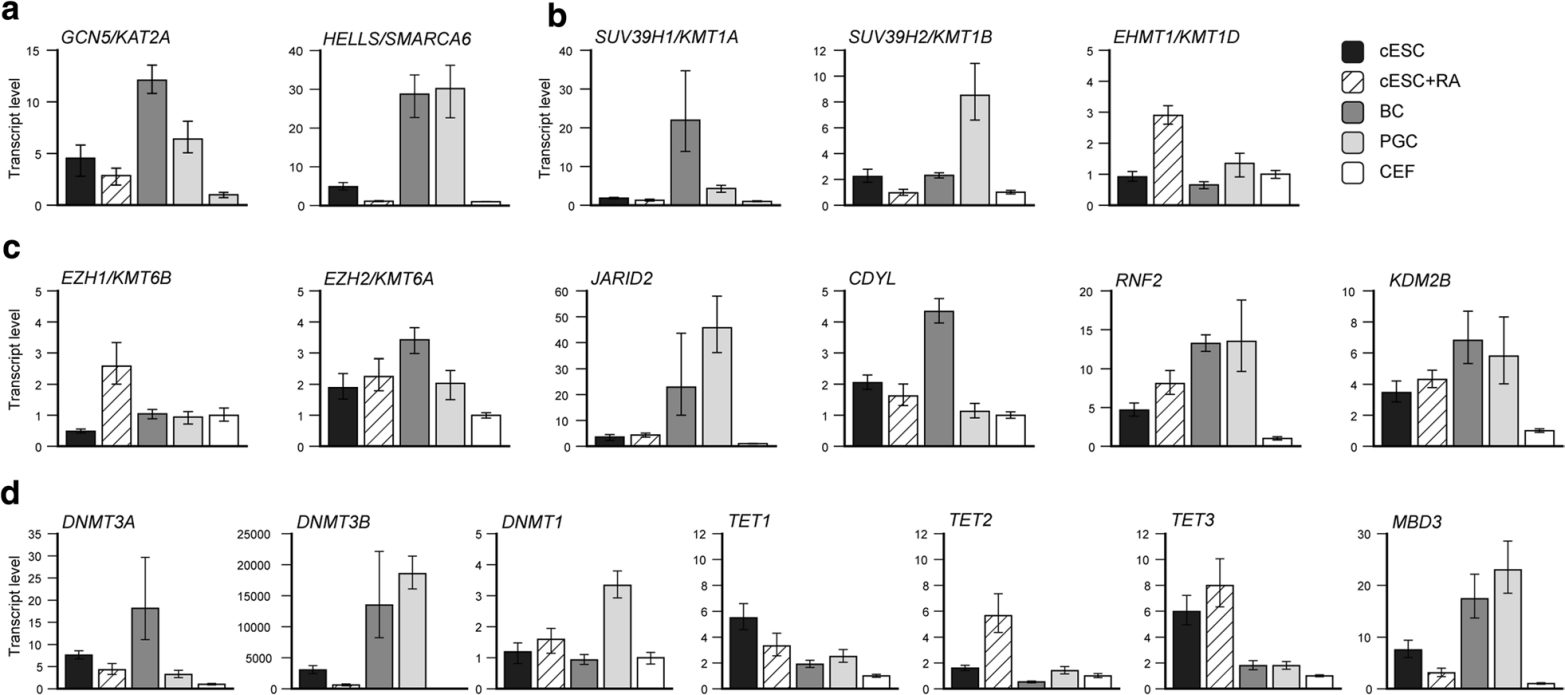
Expression of chromatin modifiers in chicken ESCs, RA-differentiated ESCs, BCs, PGCs and CEFs. **a** Histone acetylation and chromatin-remodelling factors. **b** H3K9 methylation. **c** PcG members. **d** Cytosine methylation modifiers and readers. Transcript levels were measured by RT-qPCR and normalised to levels in CEFs using the *RSP17* gene as an internal control. Means with 95 % confidence interval are represented for three technical qPCR replicates of a representative experiment

Among the genes encoding HATs, *GCN5/KAT2A* was strongly expressed in chicken ESCs compared to CEF (Fig. 7a). Taken as a whole, HAT gene expression levels were lower in ESCs than in BCs, but HDAC gene expression levels were similar (see Additional file 6). Genes encoding histone methyltransferases associated with active chromatin modifications and the corresponding demethylases were expressed at various levels (see Additional file 6). Their expression levels were, in general, similar or lower in undifferentiated compared to RA-differentiated ESCs, although the strongest expression levels were often observed in BCs and/or PGCs. The expression of several chromatin and nucleosome remodellers was also studied. None of the SMARC family genes was specifically highly expressed in chicken ESCs or PGCs apart from *HELLS/SMARCA6* (Fig. 7a and Additional file 6).

Trimethylation of H3K9 is catalysed by the SUV39H1/KMT1A and SUV39H2/KMT1B enzymes in mammals. *SUV39H1* was highly expressed in chicken BCs compared to other cell types, whereas *SUV39H2* was highly expressed in PGCs, consistent with the high level of H3K9me3 detected in those cells (Fig. 7b). *EHMT1/KMT1D,* which encodes the GLP1 enzyme responsible for H3K9 dimethylation, was not more expressed in pluripotent cells and PGCs than in CEF but was induced by RA differentiation of ESCs (Fig. 7b). The genes encoding potential H3K9 demethylases were expressed at various levels in chicken cells, but none of them were specifically more expressed in pluripotent than in differentiated cells, except for *KDM4A* and *KDM4C* which were strongly expressed in BCs and PGCs relative to ESCs (see Additional file 6).

Concerning H3K27 methylation modifiers, *EZH2* was more highly expressed in chicken ESCs, RA-differentiated ESCs, BCs and PGCs than in CEFs, whereas *EZH1* was not, with the exception of RA-differentiated ESCs (Fig. 7c). Moreover, most other components or partners of the mouse PRC2 complex were more highly expressed in ESCs than in CEFs; some of them, notably *JARID2* and *CDYL*, were highly expressed in BCs and/or PGCs (Fig. 7c and Additional file 6). The *RNF1* gene could not be found in the chicken genome annotation at the time of our analysis. *RNF2* and *KDM2B/FBXL10* were strongly expressed in chicken pluripotent cells, in good agreement with the abundance of the H2AK119ub mark in their nuclei (Fig. 7c). The genes encoding other components of PRC1 were expressed at various levels; most of them were not more highly expressed in ESCs than in CEFs, and were upregulated upon RA-induced differentiation of ESCs (see Additional file 6). The expression of *UTX/KDM6A*, encoding a potential demethylase of H3K27, was not more elevated in ESCs and BCs than in CEFs and tended to be higher in RA-differentiated ESCs (Additional file 6). Together, these data show that most PcG genes were less highly expressed in ESCs than in BCs.

The genes encoding DNMT3A and DNMT3B were extremely highly expressed in chicken ESCs in comparison with CEF and BM2 cells (Fig. 7d and data not shown). This high expression level was also observed in BCs and in PGCs, particularly for DNMT3B, as described previously [49], and was paralleled by the expression of the methyl binding domain protein MBD3 gene (Fig. 7d). Differentiation of ESCs with RA resulted in a decrease of *DNMT3* gene expression. By contrast, the *DNMT1* gene was relatively highly expressed only in PGCs. Expression of *TET1* and *TET3*, but not of *TET2*, was relatively high in ESCs compared to other cell types (Fig. 7d).

## Discussion

In this study, we obtained the first overview of chromatin epigenetic modifications in chicken stem cells by comparing ESCs and PGCs with BCs, from which they originate. Furthermore, comparisons with more differentiated cells, i.e. RA-differentiated ESCs and CEFs, allowed us to determine whether pluripotency is linked to a specific chromatin organisation, as in mouse ESCs. We identified specific and original properties of chicken ESC and PGC chromatin compared with that of mouse, notably in regard to histone H3 methylation.

### Chromatin organisation in chicken ESCs in comparison with mouse ESCs

Global chromatin organisation in mouse ESC nuclei tends to be open, as determined at the cytological level. As in mammalian ESCs, clearly delimited heterochromatin compartments, appearing as prominent DNA-dense chromocentres or electron-dense aggregates, were less prominent in undifferentiated than in differentiated chicken cells. In contrast to the mouse [15], large but well-defined H3K9me3-containing domains, corresponding to PCH, were already present in undifferentiated chicken cells and did not change much upon differentiation. PCH of chicken ESCs, which contains repetitive DNA sequences [53, 57], also harboured dense 5mC similarly to mouse ESCs, in which methylated PCH coalesces to form large clusters around the nucleoli [67]. Surprisingly, H3K27me3 was also essentially concentrated at PCH, whereas in mouse ESCs it is not enriched at PCH but is instead scattered in numerous small foci. This presence of H3K27me3 at PCH appears not to be due to culture conditions, as it can also be observed in the pregastrulating embryo cells from which ESCs are derived. H2AK119ub, deposited by the PRC1 complex, was not particularly concentrated at PCH but distributed in the whole nucleus, as in mouse ESCs. H3K9me2 distribution was also similar to that in mouse cells. Marks of open or transcriptionally active chromatin, H3K9ac, H3K4me3 and 5hmeC, were in regions of lower DNA density and were not especially enriched in pluripotent cells.

In mouse, general transcription factors and chromatin-remodelling genes are more highly expressed in ESCs than in neural precursor cells, possibly resulting in more globally open chromatin [13]. However, chromatin modifier genes, including the SMARC family genes that encode ATP-dependent chromatin-remodelling factors, were not more highly expressed in undifferentiated chicken ESCs than in RA-differentiated cells. Yet, we did observe some similarities with mouse, e.g. the expression of *BRG1* but not *BRM*, consistent with the described subunit composition of the BRG/BRAHMA-associated factors (BAF) ATP-dependent chromatin-remodelling complexes in mouse ESCs [68]. Chicken ESCs express very high levels of the *DNMT3A*, *DNMT3B* and *HELLS/SMARCA6* genes, consistent with a high DNA methylation activity prior to differentiation. This activity is likely responsible for the 5mC observed at PCH and may prepare the cells for *de novo* methylation of genes turned off at the onset of differentiation as in mouse [69–71]. *TET1* and *TET3*, but not *TET2* in contrast to the mouse model, are strongly expressed in chicken ESCs compared to BCs or to CEF. The presence of 5hmC in ESCs is consistent with a role of this modification in keeping promoters of developmental and housekeeping genes free of methylation [34, 35]. Given the inability to grow chicken ESCs in 2i medium due to the toxicity of the two chemical inhibitors on the cells, we could not investigate if chicken ESCs loose DNA methylation in these conditions as mouse ESCs do.

### High PCH enrichment in H3K27me3 is a marker of pluripotency in chicken

We found that H3K27me3 was almost exclusively concentrated at PCH in chicken ESCs, in contrast to mammalian cells, in which H3K27me3 displays a dispersed nuclear distribution [72], as a result of its presence at bivalent promoters, repressed genes and subtelomeres [73]. Moreover, we noticed that the localisation of H3K27me3 in chromatin was dynamic during chicken early development and cell differentiation. In chicken BCs, H3K27me3 was concentrated at PCH, but additional small foci could often be seen in the nucleoplasm; in more differentiated embryonic tissues, H3K27me3 was present at numerous loci, primarily in euchromatic regions of the nucleus, and nearly absent from heterochromatin. In RA-differentiated ESCs, the modification was still present at PCH, but was more widespread in the nucleoplasm than in undifferentiated ESCs, an intermediate pattern between those of undifferentiated and differentiated embryonic cells. Localisation of H3K27me3 at PCH has been observed in mouse ESCs, but only when deposition of heterochromatin marks is perturbed [33, 74, 75]. However, the presence of H3K27me3 in constitutive heterochromatin has been described in highly pluripotent mouse cells. Indeed, in early preimplantation embryos, paternal constitutive heterochromatin transiently contains H3K27me3 [25]. Furthermore, mouse ESCs harbour higher levels of H3K27me3 in satellite chromatin and lower levels of this PTM at many other genomic locations including promoters when they are grown in 2i conditions than when they are grown with serum [28]. The low level of nuclear H3K27me3 (aside from PCH) in chicken ESCs suggests that these cells are closer to a potential naive state than BCs. Therefore, we propose that high enrichment of H3K27me3 at PCH is a marker of pluripotency in chicken cells.

### Interplay between H3K27 methylation and other repressive marks

In mammals, DNA methylation is thought to be relatively independent of, and even to antagonise, the H3K27me/PcG pathway, which may assemble repressive chromatin by default [30–33]. When canonical constitutive heterochromatin harbouring H3K9 trimethylation and 5mC DNA methylation is lost at PCH, it is replaced by H3K27me3 and PcG-based heterochromatin [25, 33, 74, 75]. In chicken ESCs and in BCs, we observed abundant H3K27me3 at PCH which also harbours the canonical constitutive heterochromatin modifications, i.e. 5mC and H3K9me3. Thus, the H3K27me3 and H3K9me3 repressive pathways are not mutually exclusive in chromatin domains of chicken ESCs. Nonetheless, the antagonism may be settled later during differentiation, as H3K27me3 is progressively lost form PCH retaining H3K9me3 and 5mC. The PCH epigenetic profile of chicken ESCs is not equivalent to that of mouse ESCs in which DNA methylation is depleted [33], because ubiquitination of H2AK119 does go along with trimethylation of H3K27 in this case. The interplay between the different epigenetic marks and the chromatin modifiers which settle and read them may thus follow different rules in chicken than in mouse.

### Unique chromatin signature of chicken PGCs

In the mouse, epigenetic reprogramming of the germ cells specified in the late epiblast occurs when they migrate and settle in the gonads [4, 5]. Loss of dimethylation of H3K9 and DNA methylation, concomitant with an enhancement of H3K27 and H3K4 trimethylation and histone acetylation, occurs at E8.5. Subsequently, the marks of the different chromatin types (H3K9me3, H3K27me3, chromocentres and H3K9ac) are transiently lost, and finally the cells regain H3K9me3 and H3K27me3 around E12.5. In avian species, the germ cells are segregated from somatic cells in the epiblast very early, during the first cleavages before the egg is laid [76, 77]. PGCs migrate through the vascular system to colonise the developing gonads. After isolation from embryonic blood and long-term culture, these PGCs had a unique chromatin conformation. Indeed, their nuclei were even less rich in heterochromatin than chicken ESC nuclei, as demonstrated by the absence of the perinuclear heterochromatin layer usually detectable by electron microscopy. However, the global level of H3K9me3, typical of constitutive heterochromatin, was higher in PGCs than in chicken ESCs. This is probably favoured by the high expression of the gene encoding the SUV39H2/KMT1B histone methyltransferase, one of the chromatin-related genes that are differently expressed in PGCs relative to BCs. On the contrary, the level of H3K27me3 was lower in PGCs than in chicken ESCs, and, most importantly, the nuclear distribution of this PTM was strikingly different, with H3K27me3 only detectable at one large spot similar to ESC chromocentres. H21K119ub was also present at much fewer loci in PGCs than in other cell types. However, expression of PcG components expression was not reduced, and *JARID2* was quite highly expressed, possibly in connection with its role in promoting PRC2 activity at loci devoid of H3K27me3 [78]. 5mC formed numerous foci, whereas the 5hmC level was quite low, suggesting that the repressive chromatin in PGCs was mostly based on H3K9 and DNA methylation rather than H3K27me3. The importance of DNA methylation in PGC epigenetics was also suggested by the strong expression of *DNMT1*, *DNMT3B* and *HELLS/SMARCA6*, involved in the maintenance of methylation patterns, and of *MBD3*, which encodes a methylation reader. This original combination of H3K9me3-enriched heterochromatin and very low and confined H3K27me3 accumulation does not match any of the previously described stages of the epigenetic reprogramming of mouse PGCs.

### Dynamics of repressive chromatin epigenetic modifications during chicken early development

**Fig. 8:**
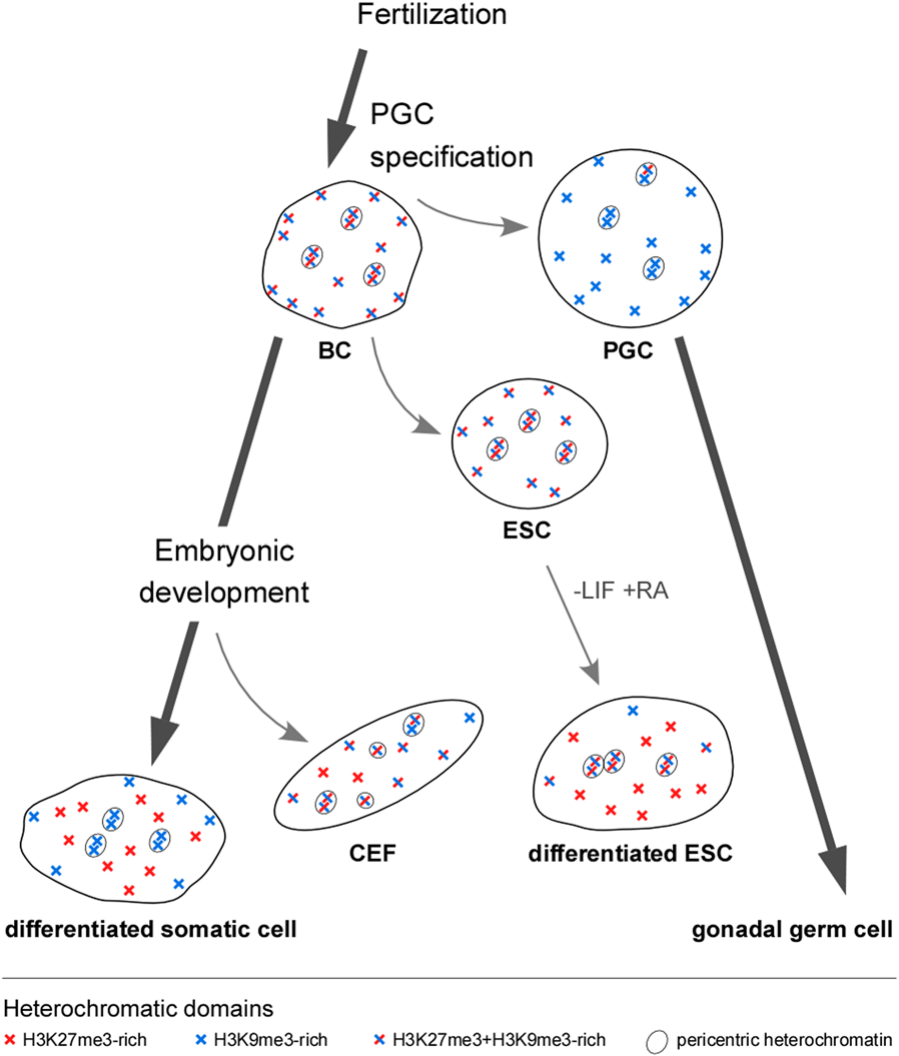
Schematic representation of the dynamics of H3K27me3 and H3K9me3-rich domains in chicken cells. The distributions of the heterochromatic domains in nuclei of BCs, ESCs, RA-differentiated ESCs, PGCs, CEFs and somatic differentiated cells are represented. A decrease of H3K27me3 enrichment at pericentric heterochromatin and an increase at other loci is observed upon in vivo and as well as in vitro differentiation

H3K27me3 concentration at PCH was high in BCs and decreased with cell differentiation until it was not enriched there any more in differentiated embryonic tissues. Concomitantly, and without loss of total H3K27me3, the appearance of numerous H3K27me3 small foci indicated the establishment of this mark at new loci [20]. Of all studied cell types, ESCs had the highest enrichment at H3K27me3 at PCH relative to other genome loci. Therefore, we propose that H3K27me3 is confined to PCH in pluripotent chicken cells, and that commitment to a somatic lineage induces its shift to other, developmentally regulated loci controlled by PcG proteins (Fig. 8). H3K27me3-based repression may partially replace H3K9me3-based repression, given that differentiation induces a decrease of H3K9me3 global level. PGCs follow a different epigenetic path in which H3K9me3 prevails over H3K27me3. Future mapping of histone PTMs and DNA methylation in the genomes of ESCs, PGCs and of the embryo will help to determine how the different epigenetic modifications control pluripotency and developmentally regulated genes during chicken early development.

## Conclusions

We showed that the main chromatin epigenetic modifications linked to active and inactive chromatin, as defined in mammals, are conserved in differentiated chicken cells. Nevertheless, in sharp contrast with mouse cells, chicken ESCs and BCs have high levels of H3K27me3 at PCH. It remains unclear whether this enrichment has a functional role in PCH maintenance or is simply a sink or a reservoir for H3K27me/PcG components to allow the expression of a prompt differentiation programme. Although PGCs are related to BCs, their epigenome differs from that of ESCs, especially in regard to their low level of H3K27me3. This overview of chromatin epigenetics during the early steps of chicken development suggests potential new functions for already well-studied histone PTMs, illustrating the value of considering multiple species in studying the roles of chromatin epigenetics during vertebrate development.

## Methods

### Embryo, cells and tissue

Chicken blastodermal cells were obtained from stage X-XII embryos [60] from JA57 strain. The chicken embryonic stem cells were established and amplified for less than 40 passages on inactivated STO feeder cells in proliferative medium containing cytokines and growth factors as described [61, 65]. Proliferating ESC were induced to differentiate by retinoic acid treatment at 5.10^−7^ M for 5 days in medium without cytokines. Long-term cultured primordial germ cells were derived from stage 19–21 HH [79] embryonic blood and maintained as described [64]. Primary chicken embryonic fibroblasts were prepared from 11- to 12-day-old embryos according to the standard protocols [80], maintained for 10 passages before being used as a somatic cell control. Mesonephros tissue was dissected from 9-day-old embryos. Mouse ESCs and EpiSC were provided by Alice Jouneau (INRA Jouy, France) [81] and Sylvie Rival-Gervier (Inserm U1208, INRA USC1361). The non-tumorigenic BM2 monocytic cell line was grown as described [82] using DMEM/F12 as basal medium instead of BT88, and used as a proliferative progenitor cell.

### Electron-transmitted microscopy

Cells were fixed in 2 % glutaraldehyde, washed three times in saccharose 0.4 M/0.2 M Na Cacodylate-HCl pH 7.4 for 1 h at 4 °C and postfixed with 2 % OsO4/0.3 M Na Cacodylate-HCl pH 7.4 for 1 h at 4 °C. They were dehydrated with an increasing ethanol gradient (5 min in 30, 50, 70, 95 %, and three times for 10 min in absolute ethanol). Impregnation was performed with Epon A (50 %) plus Epon B (50 %) plus DMP30 (1.7 %). Inclusion was obtained by polymerisation at 60 °C for 72 h. Ultrathin sections approximately 70-nm thick were cut on a Reichert ultracut E ultramicrotome (Leica), mounted on 200 mesh copper grids coated with 1:1000 polylysine, stabilised for 1 day at RT and contrasted with uranyl acetate and lead citrate. Sections were examined with a Jeol 1400JEM (Tokyo, Japan) transmission electron microscope equipped with a Orius 600 camera and Digital Micrograph.

### Fluorescence immunodetection

Embryos were washed in phosphate-buffered saline (PBS), fixed in 4 % paraformaldehyde (PFA) in PBS for 30 min at room temperature (RT) and washed three times with PBS. They were pre-embedded in 2 % low-gelling agarose and equilibrated in 30 % sucrose before being embedded in O.C.T (CellPath) and snap frozen in liquid nitrogen. Cryosections about 16-μm thick were prepared using a cryo-microtome (Leica) and deposited on Superfrost Plus slides and stored at −80 °C until use. Chicken ESC and CEF were grown on gelatinised glass coverslips, washed with PBS, fixed in 4 % PFA for 10 min at RT and washed three times in PBS. PGCs were resuspended in PBS and cytospun onto Superfrost Plus slides before fixation in the same conditions. Immunostaining was done in the same conditions for all sample types. Cells or tissue sections were permeabilised with 0.5 % Triton X-100 in PBS for 30 min at RT, washed with PBS and saturated with 2 % BSA in PBS for 1 h. They were incubated with the primary antibody overnight at 4 °C in the blocking solution (dilution 1:500). Peptide competition was performed by incubating the blocking peptide (five time excess to primary antibody by weight) in the blocking solution for 1 h before application onto cells. After three washes with 0.1 % Tween-20 in PBS at RT (10 min each), incubation with the secondary antibody was performed for 1 h in the blocking solution at RT, followed by two washes (5 min each) with 0.1 % Tween-20 in PBS and one wash with PBS at RT. DNA was counterstained for 30 min at RT using 1 μM TO-PRO-3 (Molecular Probes) in PBS. After a brief wash in PBS, samples were mounted with SlowFade Gold antifade reagent (Invitrogen). For the detection of 5mC and 5hmC, RNA was digested after permeabilisation by 100 μg/mL RNase A in PBS for 1 h at 37 °C, in order to use propidium iodide (PI) for DNA counterstaining. Acid-induced epitope unmasking was performed by denaturation in 4 N HCl for 1 h at 37 °C and washed in 100 mM Tris–HCl pH 8 and PBS. For undifferentiated chicken ESCs, tryptic digestion was required to detect 5mC signal, probably because accessibility to methylated DNA in these cells may be hindered by the association of proteins. The acid-induced epitope unmasking was shortened to 10 min and followed by a trypsin digestion performed with 0.25 % trypsin (ThermoFisher Scientific) for 45 s at 37 °C and blocked by 10 % serum in PBS [83]. Immunodetection was then performed as above except that DNA counterstaining was done in 10 μM PI. The following primary antibodies were used at 1/500 dilution: anti-H3K9me3 (#39,161), anti-H3K27me2/3 (#39,538), anti-H3K9me2 (#39,683), anti-5-hydroxymethylcytosine (#39,769), all from Active Motif; anti-H3K27me3 (#07499) from Millipore; anti-H3K4me3 (#ab8580) and anti–H3K9ac (#ab61231) from Abcam; anti-H2AK119ub (#8240) from Cell Signalling; anti-5-methylcytidine (#BI-MECY-0100) from Eurogentec; CREST antibody (#HCT-0100) from Immunovision. The anti-rabbit CENP-T antibody was a gift from T. Fukagawa (National Institute of Genetics, Mishima, Japan) and was used at 1/1000 dilution. The H3K27me3 peptide was purchased from Diagenode (#C16000069). Secondary antibodies purchased from Jackson Immunoresearch were used at 1/500 dilution. They were anti-rabbit IgG-Alexa Fluor^®^ 488 (111-545-003), anti-rabbit IgG-Cy3 (111-166-003), anti-mouse IgG-Cy3 (#115-165-003), anti-mouse IgG-FITC (#115-095-003) and anti-human-TRITC (#709-025-149).

Images acquisition of fluorescently labelled nuclei was performed using a Leica DM 6000 CS or TCS SPEII confocal laser-scanning microscope equipped with a ×63/1.4 NA oil immersion objective in sequential scanning mode. ImageJ [84] was used for image processing and drawing of intensity plots.

### RNA extraction and RT-qPCR

Total RNAs from embryos and cultured cells were extracted using the RNeasy kit (Qiagen) and TRIzol reagent (Life Technologies), respectively, according to the manufacturer’s recommendations. Reverse transcription and quantitative PCR on cDNAs were performed as described previously [62]. The comparative ΔCt method of the StepOne Plus TM software was used to obtain the RQ value for each sample, with *RSP17* gene as internal control as described [65, 66]. Primers are listed in Additional file 7: Table S1. Gene expression, initially analysed using commercially annotated microarrays [62], was checked by RT-qPCR on at least two RNA samples corresponding to two other different passages of the most homogeneous cell isolate. The most remarkable results were confirmed on the other ESC isolates.

### Histone extraction, Western blot analysis and quantification

Histones were extracted form embryos and cell pellets by acid extraction [85], resuspended in water and quantified by the Coomassie Protein Assay kit (Thermo). Three microgram of histones was diluted in SDS-loading buffer and run on a 12 % SDS-polyacrylamide gel electrophoresis, followed by Western blotting onto Hybond ECL membrane (Amersham) according to the standard protocols. Membranes were saturated with 3 % skimmed milk powder in Tris-buffered saline with Tween (TBST) and incubated overnight at 4 °C with the primary antibody diluted in the same buffer. The primary antibodies were the same as above, with the addition of anti-Histone H3 C-terminal (#07-690, Millipore), and were diluted 1/2000, excepted for anti-Histone H3 C-terminal, diluted 1:50,000. They were washed with TBST and incubated 1 h at RT with the appropriate peroxidase-conjugated secondary antibody. The secondary antibodies were anti-mouse-peroxidase (#115-035-003) and anti-rabbit-peroxidase (#115-036-003) from Jackson Immunoresearch, diluted 1:10,000. Signal detection was performed using the Clarity ECL kit (Biorad) and Biorad Chemidoc MP imaging system. Membranes were then stripped according to the manufacturer’s protocol and re-probed with the antibody against histone H3 as a loading control. Integrated pixel intensity was measured with ImageJ [84] for each band and the respective background signal was subtracted. Signals were normalised to the loading control (histone H3), and the fold difference to the control cell type (CEF) was calculated.

### Genomic DNA extraction, dot blot analysis and quantification

Cell pellets and embryos were resuspended in 400 μL of lysis buffer (10 mM Tris–HCl pH 8, 100 mM EDTA, 0.5 % SDS). RNAs were digested by adding 80 μg of RNAse A and incubating for 1 h at 37 °C. Proteins were digested for 3 h at 37 °C with 40 μg of Proteinase K and extracted by phenol–chloroform-isoamyl alcohol. DNA was ethanol-precipitated with sodium acetate 0.3 M, washed with 70 % ethanol, dried and resuspended in 10 mM Tris–HCl pH 8. DNA was digested with SpeI (New England Biolabs) to allow precise pipetting of small volumes, cleaned by phenol–chloroform-isoamyl alcohol and precipitated as above. For each sample, 125 ng of DNA denatured in 0.4 N NaOH was spotted onto Hybond N + membranes (GE Healthcare Lifesciences) in triplicate. Once dried, the membranes were saturated and incubated with anti-5-methylcytidine diluted 1:1000 or anti-5-hydroxymethylcytosine diluted 1:10,000. Secondary antibodies incubations and signal detections were performed as for Western blots.

## Additional files

10.1186/s13072-016-0056-6 Nuclear distribution of chromocentres in the nuclei of interphase chicken ESCs. Co-immunodetection of centromeric proteins by CREST antibody (a, b) and H3K9me3 (c, d) with DNA counterstaining with TO-PRO-3 (e, f) in nuclei of ESCs and RA-differentiated ESCs. Single confocal images of representative nuclei are shown. Scale bar 5 μm.
10.1186/s13072-016-0056-6 Nuclear distribution of chromatin modifications in E14Tg2A mouse ESCs (A) Co-immunodetection of H3K9me3 (a) and H3K27me2/3 (b), and DNA counterstaining with TO-PRO-3 (c). Overlay of H3K9me3 (magenta), H3K27me2/3 (green) is shown in (d). (B) Co-immunodetection of H2AK119ub (a) and H3K27me2/3 (b), and DNA counterstaining with TO-PRO-3 (c) in nuclei of E14Tg2A ESCs cultivated with serum. Overlay of H2AK119ub (green) and H3K27me2/3 (magenta) is shown in (d). (C) Co-immunodetection of H3K9me2 (a) and H3K27me3 (b), and DNA counterstaining with TO-PRO-3 (c). Overlay of H3K9me2 (green) and H3K27me3 (magenta) is shown in (d). (D) Immunodetection of 5-methylcytosine (a) and DNA counterstaining by propidium iodide (a) (E) Immunodetection of 5-hydroxymethylcytosine (a) and DNA counterstaining by propidium iodide (b). Single confocal sections of representative nuclei are shown. Scale bar 5 μm.
10.1186/s13072-016-0056-6 Control experiments for H3K27me3 immunodetection in chicken cells. (A) Co-immunodetection of H3K27me2/3 (a–d) and H3K9me3 (e–h), and DNA counterstaining by TO-PRO-3 (i–l) in nuclei of ESCs and RA-differentiated ESCs. H3K27me3 competitor peptide was incubated with the H3K27me2/3 antibody in (b, d, f, h, j and l) as a negative control for the specificity of the anti-H3K27me2/3 antibody. (B) Immunodetection of H3K27me3 (a–e) and DNA counterstaining by TO-PRO-3 (f–j) in nuclei of ESCs, RA-differentiated ESCs, BCs, PGCs and CEFs. Single confocal sections of representative nuclei are shown. Scale bar 5 μm.
10.1186/s13072-016-0056-6 Nuclear distribution of histone post-translational modifications of active chromatin in chicken ESCs, RA-differentiated ESCs, BCs, PGCs and CEFs. (A) Immunodetection of H3K4me3 (a–e) and DNA counterstaining with TO-PRO-3 (f–j) in nuclei of ESCs, RA-differentiated ESCs, BCs, PGCs and CEFs. Overlay of H3K4me3 (green) and TO-PRO-3 (magenta) is shown below (k–o). (B) Immunodetection of H3K9ac (a–e) and DNA counterstaining with TO-PRO-3 (f–j) in nuclei of ESCs, RA-differentiated ESCs, BCs, PGCs and CEFs. Overlay of H3K9ac (green) and TO-PRO-3 (magenta) is shown below (k–o). Single confocal sections of representative nuclei are shown. Scale bar 5 μm.
10.1186/s13072-016-0056-6 Western and dot blot supplementary data. (A) Western blot images of H3 post-translational modifications detection in chicken ESCs, RA-differentiated ESCs, BCs, PGCs and CEFs. 3 μg of purified histones were analysed by SDS-PAGE followed by blotting and immunodetection using an antibody against a modification (upper panels), and an antibody against H3 after stripping of the membrane (lower panels). Molecular weights are indicated in kDa. (C) Global levels of 5-methylcytosine and 5-hydroxymethylcytosine in mouse cells. 5mC and 5hmC levels were quantified by dot blot analysis of 125 ng of denatured genomic DNA from mouse ESC cultured in 2i or serum medium and from mouse epiblast stem cells (EpiSCs); pBSK plasmid was used as a negative control. Error bars indicate the standard deviation of the mean signal for three technical replicates of one representative experiment.
10.1186/s13072-016-0056-6 Expression of chromatin modifiers in chicken ESCs, RA-differentiated ESCs, BCs, PGCs and CEFs. (A) Histone acetylation. (B) Chromatin-remodelling factors. (C) H3K4, H3K36 and arginine methylation. (D) H3K9 methylation. (E) PcG members. Transcript levels were measured by RT-qPCR and normalised to levels in CEFs using the *RSP17* gene as an internal control. Means with 95 % confidence interval are represented for three technical qPCR replicates of a representative experiment.
10.1186/s13072-016-0056-6 Oligonucleotides used for RT-qPCR gene expression analysis. Gene IDs are accession numbers from the NCBI database. Sequences are given 5′ to 3′.

## Abbreviations

5mC: 5-methylcytosine
5hmC: 5-hydroxymethylcytosine
BC: blastodermal cell
CEF: chicken embryonic fibroblast
ESC: embryonic stem cell
PcG: polycomb group
PCH: pericentric heterochromatin
PGC: primordial germ cell
PRC: polycomb repressive complex
