# Supplementary figures and images for: Chicken embryonic stem cells and primordial germ cells display different heterochromatic histone marks than their mammalian counterparts
Source: Epigenetics Chromatin. 2016 Feb 10;9:5. doi: 10.1186/s13072-016-0056-6 (PMC4748481; doi:10.1186/s13072-016-0056-6)

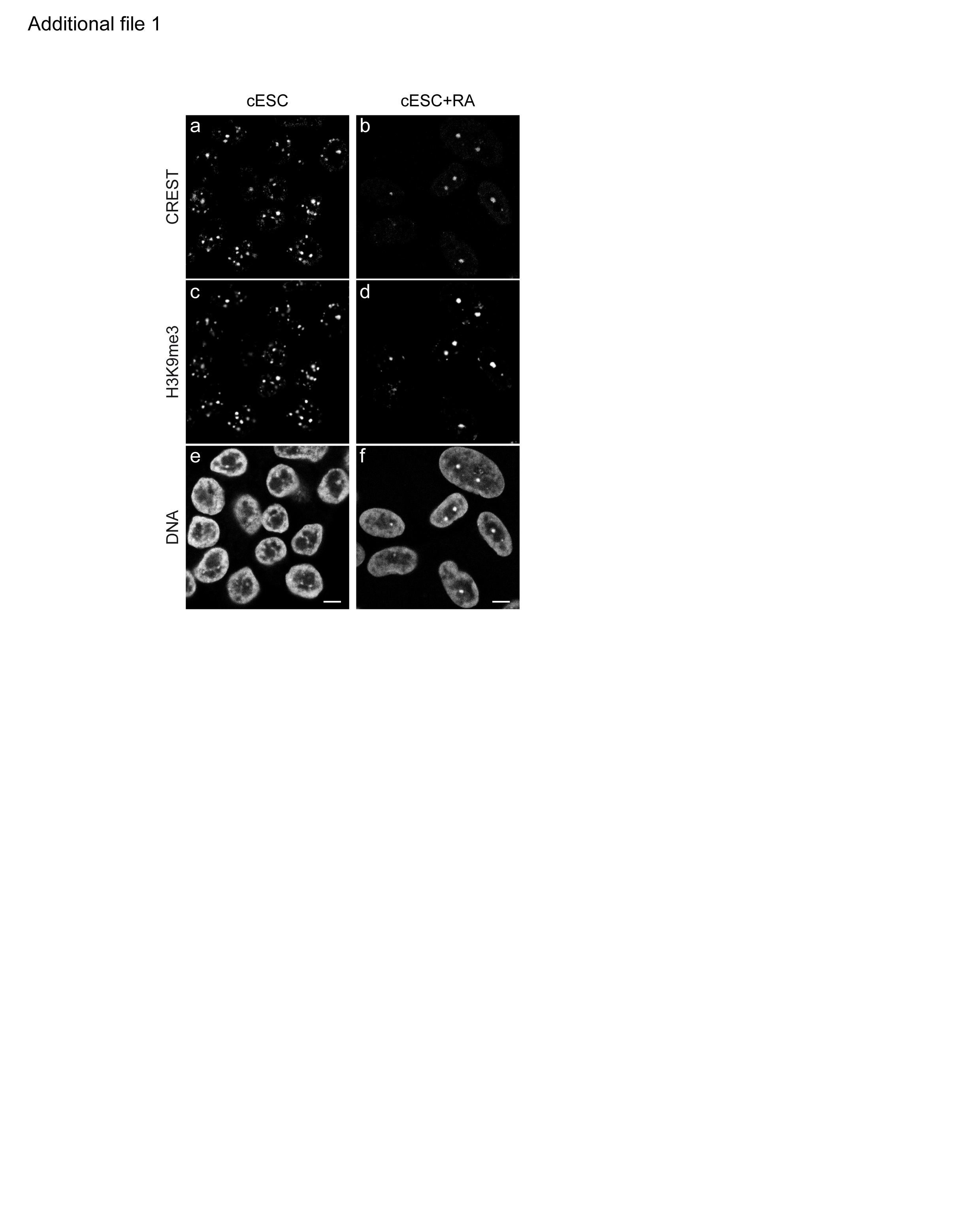

Supplement: Supplementary file 1 — 10.1186/s13072-016-0056-6 Nuclear distribution of chromocentres in the nuclei of interphase chicken ESCs. Co-immunodetection of centromeric proteins by CREST antibody (a, b) and H3K9me3 (c, d) with DNA counterstaining with TO-PRO-3 (e, f) in nuclei of ESCs and RA-differentiated ESCs. Single confocal images of representative nuclei are shown. Scale bar 5 μm. [file 13072_2016_56_MOESM1_ESM.tif]

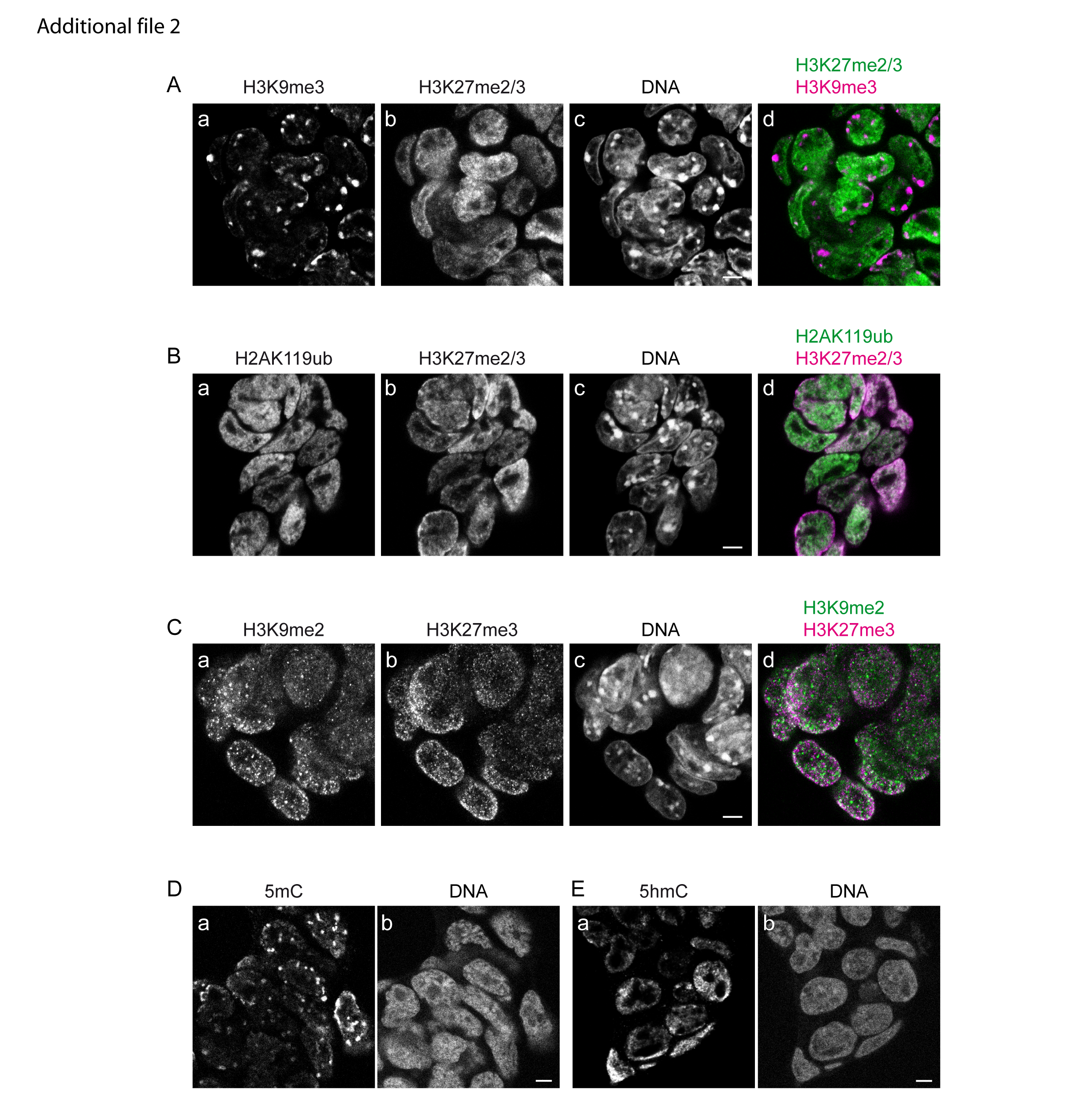

Supplement: Supplementary file 2 — 10.1186/s13072-016-0056-6 Nuclear distribution of chromatin modifications in E14Tg2A mouse ESCs (A) Co-immunodetection of H3K9me3 (a) and H3K27me2/3 (b), and DNA counterstaining with TO-PRO-3 (c). Overlay of H3K9me3 (magenta), H3K27me2/3 (green) is shown in (d). (B) Co-immunodetection of H2AK119ub (a) and H3K27me2/3 (b), and DNA counterstaining with TO-PRO-3 (c) in nuclei of E14Tg2A ESCs cultivated with serum. Overlay of H2AK119ub (green) and H3K27me2/3 (magenta) is shown in (d). (C) Co-immunodetection of H3K9me2 (a) and H3K27me3 (b), and DNA counterstaining with TO-PRO-3 (c). Overlay of H3K9me2 (green) and H3K27me3 (magenta) is shown in (d). (D) Immunodetection of 5-methylcytosine (a) and DNA counterstaining by propidium iodide (a) (E) Immunodetection of 5-hydroxymethylcytosine (a) and DNA counterstaining by propidium iodide (b). Single confocal sections of representative nuclei are shown. Scale bar 5 μm. [file 13072_2016_56_MOESM2_ESM.tif]

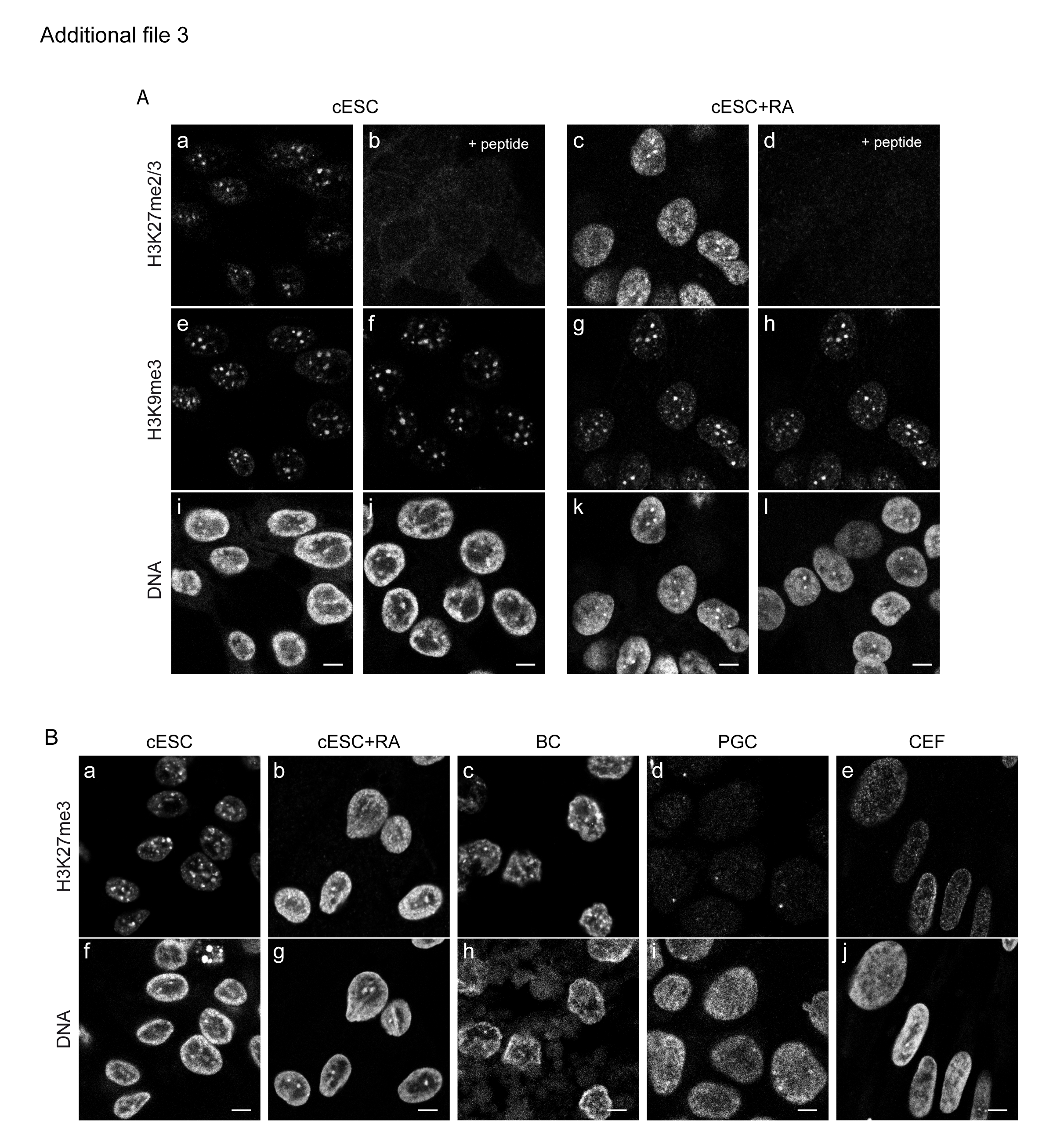

Supplement: Supplementary file 3 — 10.1186/s13072-016-0056-6 Control experiments for H3K27me3 immunodetection in chicken cells. (A) Co-immunodetection of H3K27me2/3 (a–d) and H3K9me3 (e–h), and DNA counterstaining by TO-PRO-3 (i–l) in nuclei of ESCs and RA-differentiated ESCs. H3K27me3 competitor peptide was incubated with the H3K27me2/3 antibody in (b, d, f, h, j and l) as a negative control for the specificity of the anti-H3K27me2/3 antibody. (B) Immunodetection of H3K27me3 (a–e) and DNA counterstaining by TO-PRO-3 (f–j) in nuclei of ESCs, RA-differentiated ESCs, BCs, PGCs and CEFs. Single confocal sections of representative nuclei are shown. Scale bar 5 μm. [file 13072_2016_56_MOESM3_ESM.tif]

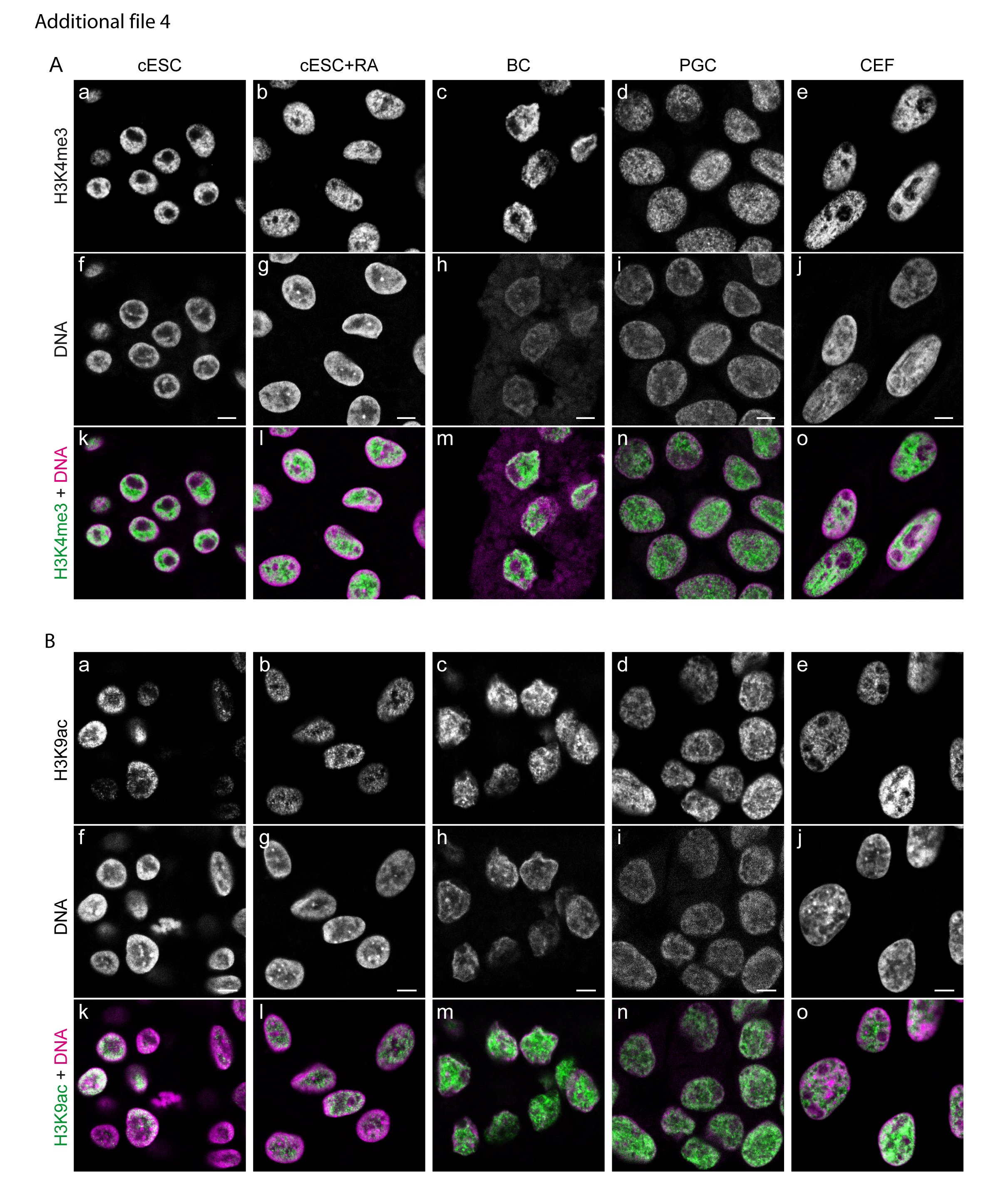

Supplement: Supplementary file 4 — 10.1186/s13072-016-0056-6 Nuclear distribution of histone post-translational modifications of active chromatin in chicken ESCs, RA-differentiated ESCs, BCs, PGCs and CEFs. (A) Immunodetection of H3K4me3 (a–e) and DNA counterstaining with TO-PRO-3 (f–j) in nuclei of ESCs, RA-differentiated ESCs, BCs, PGCs and CEFs. Overlay of H3K4me3 (green) and TO-PRO-3 (magenta) is shown below (k–o). (B) Immunodetection of H3K9ac (a–e) and DNA counterstaining with TO-PRO-3 (f–j) in nuclei of ESCs, RA-differentiated ESCs, BCs, PGCs and CEFs. Overlay of H3K9ac (green) and TO-PRO-3 (magenta) is shown below (k–o). Single confocal sections of representative nuclei are shown. Scale bar 5 μm. [file 13072_2016_56_MOESM4_ESM.tif]

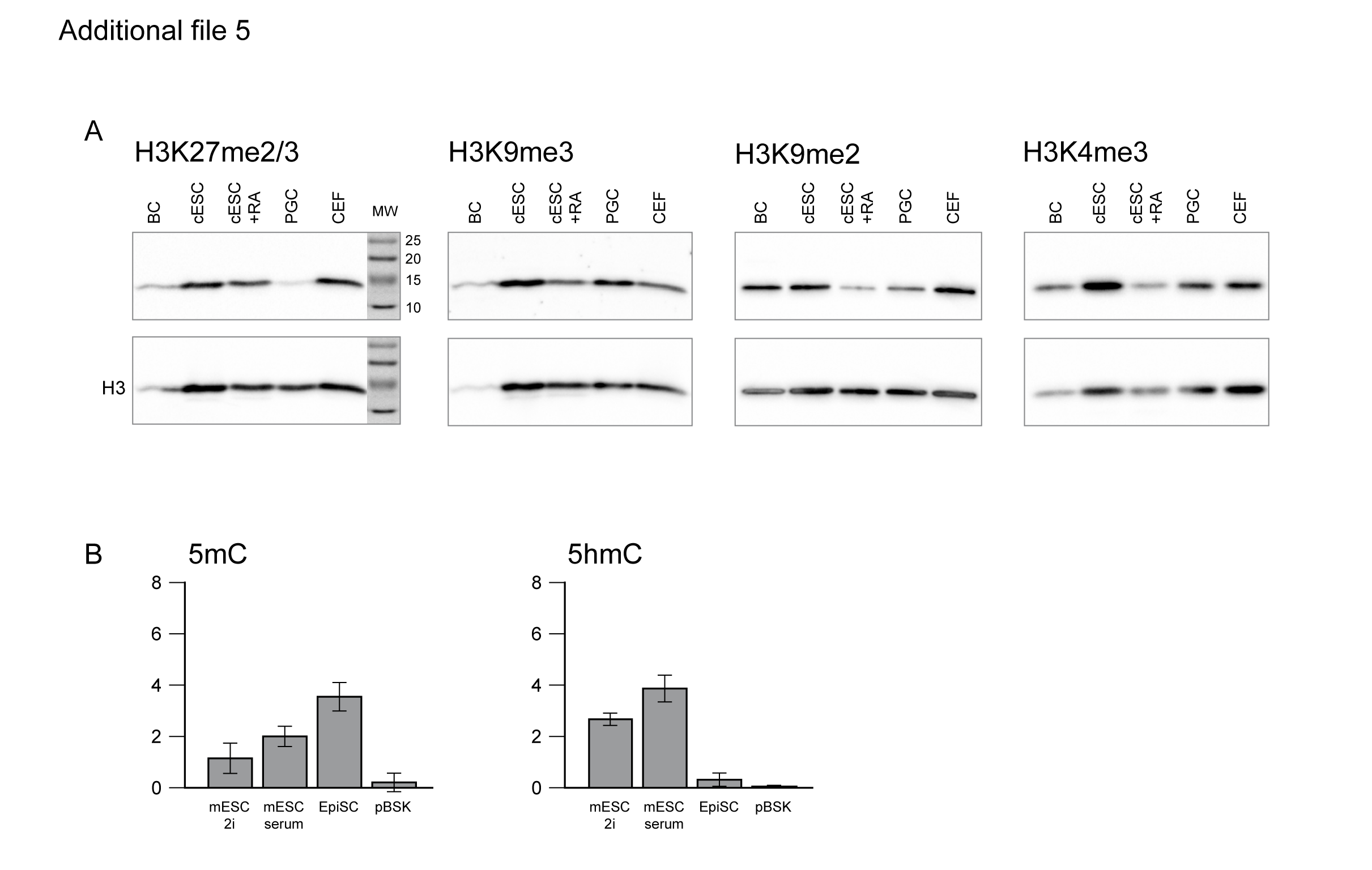

Supplement: Supplementary file 5 — 10.1186/s13072-016-0056-6 Western and dot blot supplementary data. (A) Western blot images of H3 post-translational modifications detection in chicken ESCs, RA-differentiated ESCs, BCs, PGCs and CEFs. 3 μg of purified histones were analysed by SDS-PAGE followed by blotting and immunodetection using an antibody against a modification (upper panels), and an antibody against H3 after stripping of the membrane (lower panels). Molecular weights are indicated in kDa. (C) Global levels of 5-methylcytosine and 5-hydroxymethylcytosine in mouse cells. 5mC and 5hmC levels were quantified by dot blot analysis of 125 ng of denatured genomic DNA from mouse ESC cultured in 2i or serum medium and from mouse epiblast stem cells (EpiSCs); pBSK plasmid was used as a negative control. Error bars indicate the standard deviation of the mean signal for three technical replicates of one representative experiment. [file 13072_2016_56_MOESM5_ESM.tif]

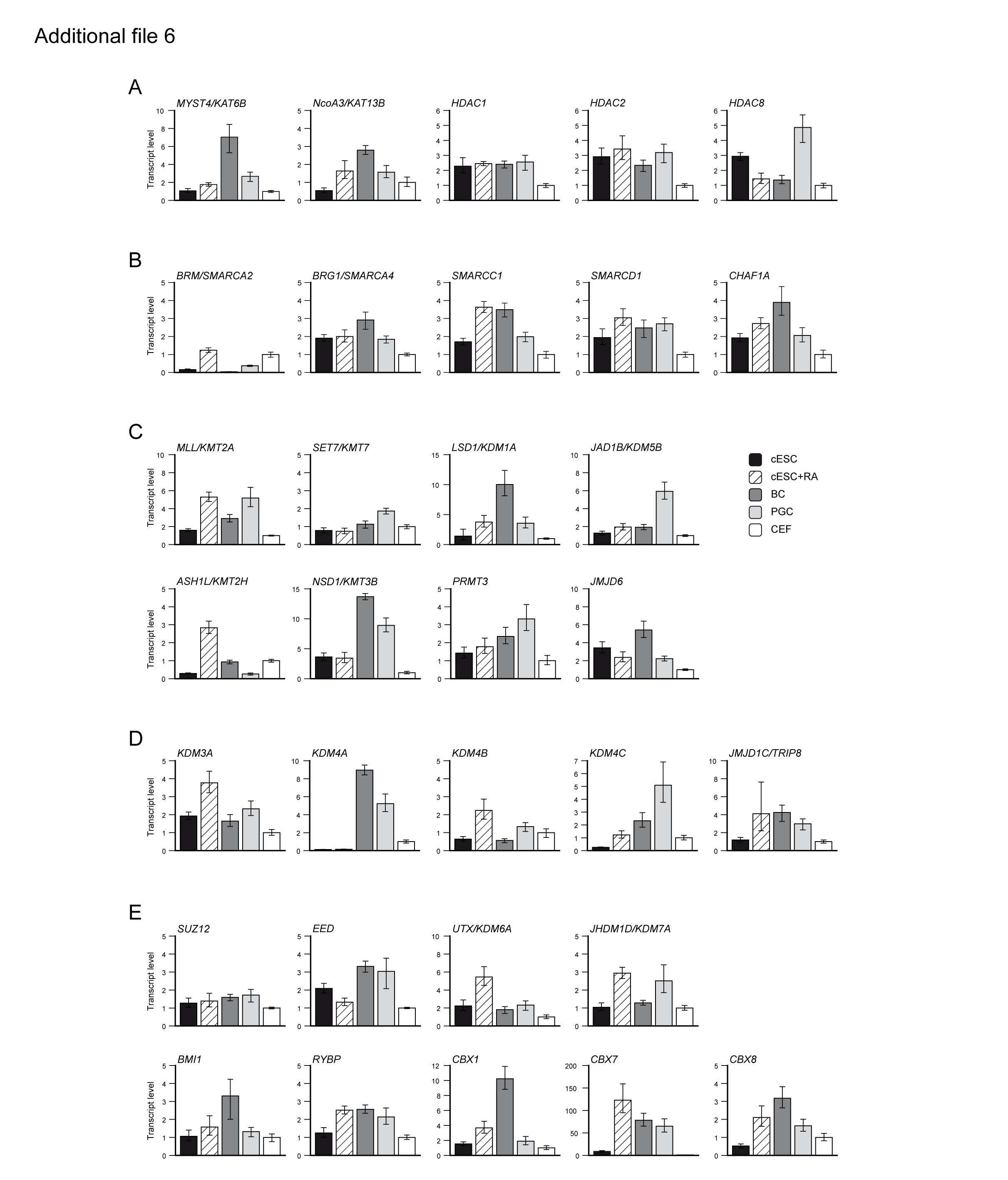

Supplement: Supplementary file 6 — 10.1186/s13072-016-0056-6 Expression of chromatin modifiers in chicken ESCs, RA-differentiated ESCs, BCs, PGCs and CEFs. (A) Histone acetylation. (B) Chromatin-remodelling factors. (C) H3K4, H3K36 and arginine methylation. (D) H3K9 methylation. (E) PcG members. Transcript levels were measured by RT-qPCR and normalised to levels in CEFs using the RSP17 gene as an internal control. Means with 95 % confidence interval are represented for three technical qPCR replicates of a representative experiment. [file 13072_2016_56_MOESM6_ESM.tif]
