# Supplementary material for: Chicken embryonic stem cells and primordial germ cells display different heterochromatic histone marks than their mammalian counterparts
Source: Epigenetics Chromatin. 2016 Feb 10;9:5. doi: 10.1186/s13072-016-0056-6 (PMC4748481; doi:10.1186/s13072-016-0056-6)
Supplement: Supplementary file 7 — 10.1186/s13072-016-0056-6 Oligonucleotides used for RT-qPCR gene expression analysis. Gene IDs are accession numbers from the NCBI database. Sequences are given 5′ to 3′. [file 13072_2016_56_MOESM7_ESM.pdf]

Table 1

Gene name	Gene ID	Forward primer	Reverse primer
BRM/SMARCA2	396040	TGAGTTGCCATCGTGGATTA	TCCTCCTTCCCAGAATCCTT
BRG1/SMARCA4	395932	CCGCTGGAAGTACATGATTGT	TCGGATGATCAGAATGGTCTC
HELLS/SMARCA6	423750	AAGCTCTGCTGGCAACCTGTGTC	AGGAACTGCTTGGCACTGTGTCG
SMARCC1	420378	AGGAGGCAGAAGAGAACAAGG	AGCAACCAGGGACTTGATTTT
SMARCD1	426882	GACCAGACGATCATGAGGAAA	ATCTTCTAGCAGCCGTCCTTC
CHAF1A	770217	CCAGAAACCAAAGACTCCACAAG	CGACAGAGGGGAGCTAAGAC
GCN5/KAT2A	374232	CCGCTACCTGGGCTACATC	GCGCTTGCTTCCTCTCAATC
MYST4/KAT6B	423734	GATGATGGGAACTCAGCCATA	TCCAATACGCCAGTTTGGTAG
NcoA3/KAT13B	419208	CATGGATTTGTCTCTACCCACTTTC	AGCCACTGTTGTTCTGATGTTTTG
HDAC1	373961	CGGATGACCCACAACCTACT	TCTGTCAGCTCTCTGCTGGA
HDAC2	395635	GGGACATTGGTGCTGGAAAAGGC	ACAGCACTGGGCTGGTACATCTC
HDAC8	422182	CAGAAATTTGACCGCATCCT	GCCATCTTGGATAGGCACAT
MLL/KMT2A	414897	CCCTGTTCCCCTTACAGAAAC	TCCTGATCCTAAGACCCTTTGAG
SET7/ KMT7	422443	TTTTCAAAAGTAGCGGCAGAA	CAAACGGGTCATAGATGCAGT
LSD1/KDM1A	419571	GAGCGGCATGGACTGATTAAC	CAACCCAGACACACCAGAAC
JAD1B/KDM5B	421168	GGCTCAGGAGTGCAATAAGC	CTGCCAAACTCCTTTGAAGC
ASH1L/KMT2H	425064	CTGGCACAGATCTTCAAGGAG	ACATCACGACCAGTTGGAGAC
NSD1/KMT3B	416214	AAAAAGGACAGGCAGCTGAA	ATGCATGTTTCTCCGGTAGG
KDM2B	416844	GATTCTCTGGTGTTTGGTGGA	GTCTAACCACGAATCCGATGA
PRMT3	422975	AGATGTCGGAAGCAGCTCAT	ATTCCAGTTCCACAGCCAAC
JMJD6	417355	TAGAAACCTGCCTTGCTTTGA	GAGCACAAAAGATGCTTCAGG
SUV39H1/KMT1A	100857640	CTCTTCGACCTGGACTACGTG	CTGCATCTACTGGGTCCACAT
SUV39H2/KMT1B	426314	GACGAGGCCAATTCTATGACA	CCTTGATGGTTCTTGTGGAAA
EHMT1/KMT1D	417250	TGCGCTGCTGGTATGATAAG	GGATATCAGCTCGCCAACAT
KDM3A	422917	AAACGCCTTCACCAGGAGTAT	AGGCTGGATTCGTTAGCTCTC
KDM4A	424571	GGCAGATAGTCATCAGCAAGC	ACCTGTCCATCTGTCCATCTG
KDM4B	428354	GCCAAGTTTATTGCAGCTCAG	CTTGGCTGCTCTCATTTCATC
KDM4C	427231	TGAAGTGCCAGCAACGTAATTC	TCACCATGTCCCACCCTATTTC
EZH1/KMT6B	420023	AGACTGCTGGGAACAAAGACA	TCAGCTGAATCTTCCTGCAAT
EZH2/KMT6A	420784	AGGAGCTCTTCCTCCTGAATG	TTCGGTGGTGTCTTTATACGC
SUZ12	417406	ACCTCTCCGTCCACAAGAAAT	GCAATAAACCCGTGCTTCATA
EED	426381	GCAGGGTCCAGGGGTATTAT	TCATCCCTGTGCCCTTCTAC
JARID2	420839	TAAGGAGGTGGGGAGACAGTT	GCATTTTCAGTTTTGCCATGT
CDYL	420877	TTAGCAGCCAATGGTACAACC	GTGCGTGAATCCATCTTGTTT
UTX/KDM6A	418556	CCTCACCATCTTCAGCCATT	ACTTCTGCTGAGCTGGGGTA
JHDM1D/KDM7A	418110	CTGGAACACGGACCTTTGTTA	TCACCACCTACATAGCGTTCC
RING1B/RNF2	424452	CCAAGCCGTGACGAATATGAAG	GTTTCTTGCCCCTCTGTAACC
BMI1	493647	TGAAGACAGGGGAGAAGTGG	ACTCCGCAGGAACTTTCGTA
RYBP	374237	CCACCACCACCCAAGAAG	TGACGCTGGGACTGATCTC
CBX1	374237	CCAAGGAAGCCAACATCAAGTG	GCGGGTGCCAGGTTTTAG
CBX7	101752246	GACCCTCGCCTGGTAGTG	GTAGGAGGCGCTTTGGTTTG
CBX8	422084	GAGCGAGAAATGGAGCTATTCG	CTGCGGAACTCATAGGTTTTGG
DNMT3A	421991	CTGTCCCTGCTGAAAAGAGG	GCCCCACTCCTGTATGTGTT
DNMT3B	419287	GCTGAAGCCCATGATTGATT	ACCATTTGTTCTCGGGTCTG
DNMT1	770153	CTGCGCATCTGGAAGTTCTAC	GATCCTCGAAGCTCTTCGTTT
MBD3	770153	CAGCCTGTCACAAAGATCACA	AGAAGGGTCTCATCAGTGCAA
TET1	423690	GCGTCGCTGTGGCACCTTCT	CCGCGTGGGATGATTGCGGT
TET2	422540	CACAGAGCGCCCGAGTGTCG	GGGCGCAGAAGTCCAGGCAG
TET3	425829	CGGCGAGAAGGGCAAAGCCA	CCCACTTGGCGATGGGGCAG
